# Widespread detection of chlorine oxyacids in the Arctic atmosphere

**DOI:** 10.1038/s41467-023-37387-y

**Published:** 2023-03-30

**Authors:** Yee Jun Tham, Nina Sarnela, Siddharth Iyer, Qinyi Li, Hélène Angot, Lauriane L. J. Quéléver, Ivo Beck, Tiia Laurila, Lisa J. Beck, Matthew Boyer, Javier Carmona-García, Ana Borrego-Sánchez, Daniel Roca-Sanjuán, Otso Peräkylä, Roseline C. Thakur, Xu-Cheng He, Qiaozhi Zha, Dean Howard, Byron Blomquist, Stephen D. Archer, Ludovic Bariteau, Kevin Posman, Jacques Hueber, Detlev Helmig, Hans-Werner Jacobi, Heikki Junninen, Markku Kulmala, Anoop S. Mahajan, Andreas Massling, Henrik Skov, Mikko Sipilä, Joseph S. Francisco, Julia Schmale, Tuija Jokinen, Alfonso Saiz-Lopez

**Affiliations:** 1 Institute for Atmospheric and Earth System Research/Physics, Faculty of Science, University of Helsinki, 00014 Helsinki, Finland; 2 School of Marine Sciences, Sun Yat-sen University, Zhuhai, 519082 China; 3 Guangdong Provincial Key Laboratory of Marine Resources and Coastal Engineering, Zhuhai, 519082 China; 4 Aerosol Physics Laboratory, Tampere University, Tampere, FI-3720 Finland; 5 Department of Atmospheric Chemistry and Climate, Institute of Physical Chemistry Rocasolano, CSIC, Madrid, 28006 Spain; 6 Extreme Environments Research Laboratory, École Polytechnique Fédérale de Lausanne, (EPFL) Valais Wallis, Sion, Switzerland; 7 Univ. Grenoble Alpes, CNRS, INRAE, IRD, Grenoble INP, IGE, 38000 Grenoble, France; 8 Institut de Ciència Molecular, Universitat de València, P.O. Box 22085, València, 46071 Spain; 9 Instituto Andaluz de Ciencias de la Tierra, CSIC-University of Granada, Av. de las Palmeras 4, 18100 Armilla, Granada Spain; 10 Institute of Arctic and Alpine Research, University of Colorado, Boulder, CO 80309 USA; 11 Cooperative Institute for Research in Environmental Science, University of Colorado, Boulder, CO 80309 USA; 12 Physical Sciences Laboratory, National Oceanic and Atmospheric Administration, Boulder, CO 80305 USA; 13 Bigelow Laboratory for Ocean Sciences, East Boothbay, Maine, USA; 14 Laboratory of Environmental Physics, Institute of Physics, University of Tartu, Tartu, Estonia; 15 Indian Institute of Tropical Meteorology, Ministry of Earth Sciences, Pune, 411008 India; 16 Department of Environmental Science, iClimate, Aarhus University, Roskilde, Denmark; 17 Department of Earth and Environmental Sciences and Department of Chemistry, University of Pennsylvania, Philadelphia, Pennsylvania 19104 USA; 18 Climate and Atmosphere Research Centre (CARE-C), the Cyprus Institute, P.O. Box 27456, Nicosia, CY-1645 Cyprus; 19 Present Address: Department of Civil and Environmental Engineering, The Hong Kong Polytechnic University, Hong Kong, China; 20 Present Address: JH Atmospheric Instrumentation Design, Boulder, CO USA; 21 Present Address: Boulder Atmosphere Innovation Research LLC, Boulder, CO USA

**Keywords:** Atmospheric chemistry, Atmospheric chemistry

## Abstract

Chlorine radicals are strong atmospheric oxidants known to play an important role in the depletion of surface ozone and the degradation of methane in the Arctic troposphere. Initial oxidation processes of chlorine produce chlorine oxides, and it has been speculated that the final oxidation steps lead to the formation of chloric (HClO_3_) and perchloric (HClO_4_) acids, although these two species have not been detected in the atmosphere. Here, we present atmospheric observations of gas-phase HClO_3_ and HClO_4_. Significant levels of HClO_3_ were observed during springtime at Greenland (Villum Research Station), Ny-Ålesund research station and over the central Arctic Ocean, on-board research vessel Polarstern during the Multidisciplinary drifting Observatory for the Study of the Arctic Climate (MOSAiC) campaign, with estimated concentrations up to 7 × 10^6^ molecule cm^−3^. The increase in HClO_3_, concomitantly with that in HClO_4_, was linked to the increase in bromine levels. These observations indicated that bromine chemistry enhances the formation of OClO, which is subsequently oxidized into HClO_3_ and HClO_4_ by hydroxyl radicals. HClO_3_ and HClO_4_ are not photoactive and therefore their loss through heterogeneous uptake on aerosol and snow surfaces can function as a previously missing atmospheric sink for reactive chlorine, thereby reducing the chlorine-driven oxidation capacity in the Arctic boundary layer. Our study reveals additional chlorine species in the atmosphere, providing further insights into atmospheric chlorine cycling in the polar environment.

## Abstract (web summary)

Observations are reported of HClO_3_ and HClO_4_ in the atmosphere and their widespread occurrence over the pan-Arctic during spring, providing further insights into atmospheric chlorine cycling in the polar environment.

## Introduction

Active chlorine cycling has been found in the Arctic boundary layer during the springtime following polar sunrise and is acknowledged to play a key role in the depletion of surface ozone (O_3_) in this region^1–3^. Chlorine atoms (Cl) are also a strong oxidant in the polar troposphere, where the levels of hydroxyl radicals, another major atmospheric oxidant, are relatively low^4^. It is also well established that the direct reaction with Cl provides a chemical sink of methane (CH_4_) in the atmosphere^5–8^. The presence of chlorine species such as molecular chlorine (Cl_2_) and bromine monochloride (BrCl) has been reported in the Arctic, attributed to direct emissions from snowpacks^9,10^ and heterogeneous reactions of chlorine species on snow grains and airborne aerosols^11^. Upon photolysis, these chlorine species release reactive Cl atoms (1−2), which rapidly react with O_3_ to form chlorine monoxide, ClO (3)^3^. ClO is subsequently oxidized by bromine monoxide (BrO) or ClO, producing chlorine dioxide (OClO); reacting with HO_2_ to form hypochlorous acid (HOCl); or reacting with nitric oxide (NO) and nitrogen dioxide (NO_2_) to produce Cl atoms and chlorine nitrate (ClONO_2_) (as shown in reactions 4−7)^12^. Cl atoms can also degrade other hydrocarbons (RH) to generate hydrochloric acid (HCl; 8)^13^. HCl can be converted into chloride (Cl^−^) via hydrolysis on aerosol surfaces (9)^14^. Chloride can further undergo heterogeneous reactions with HOCl to produce Cl_2_ (10), which can, in turn, be photolyzed to recycle Cl atoms (1)^15^.

1 $${{{{{\mathrm{Cl}}}}}}_{2}+{hv}\to 2{{{{\mathrm{Cl}}}}}$$

2 $${{{{\mathrm{BrCl}}}}}+{hv}\to {{{{\mathrm{Cl}}}}}+{{{{\mathrm{Br}}}}}$$

3 $${{{{\mathrm{Cl}}}}}+{{{{\mathrm{O}}}}}_{3}\to {{{{\mathrm{ClO}}}}}+{{{{{\mathrm{O}}}}}}_{2}$$

4 $${{{{\mathrm{ClO}}}}}+{{{{\mathrm{BrO}}}}}\,{{{{\mathrm{or}}}}}\,{{{{\mathrm{ClO}}}}}\to {{{{\mathrm{OClO}}}}}+{{{{\mathrm{Br}}}}}\,{{{{\mathrm{or}}}} \,{{{\mathrm{Cl}}}}}$$

5 $${{{{\mathrm{ClO}}}}}+{{{{{\mathrm{HO}}}}}}_{2}\to {{{{\mathrm{HOCl}}}}}+{{{{{\mathrm{O}}}}}}_{2}$$

6 $${{{{\mathrm{ClO}}}}}+{{{{\mathrm{NO}}}}}\to {{{{\mathrm{Cl}}}}}+{{{{{\mathrm{NO}}}}}}_{2}$$

7 $${{{{\mathrm{ClO}}}}}+{{{{{\mathrm{NO}}}}}}_{2}\to {{{{{\mathrm{ClONO}}}}}}_{2}$$

8 $${{{{\mathrm{Cl}}}}}+{{{{\mathrm{CH}}}}}_{4}\,{{{{\mathrm{or RH}}}}}\to {{{{\mathrm{HCl}}}}}+{{{{\mathrm{CH}}}}}_{3}{{{{\mathrm{ or}}}}\, {{{\mathrm{R}}}}}$$

9 $${{{{\mathrm{HCl}}}}}\leftrightarrows {{{{{\mathrm{Cl}}}}}}^{-}({{{{\mathrm{aq}}}}})$$

10 $${{{{{\mathrm{Cl}}}}}}^{-}({{{{\mathrm{aq}}}}})+{{{{\mathrm{HOCl}}}}}+{{{{{\mathrm{H}}}}}}^{+}({{{{\mathrm{aq}}}}})\to {{{{{\mathrm{Cl}}}}}}_{2}+{{{{{\mathrm{H}}}}}}_{2}{{{{\mathrm{O}}}}}$$

Despite decades of research on chlorine cycling in the atmosphere, a largely unexplored aspect entails the formation of chlorine oxyacids, such as chloric (HClO_3_) and perchloric (HClO_4_) acids. The presence of atmospheric HClO_4_ was first proposed to be important in the polar stratosphere and is believed to be a missing atmospheric sink process of chlorine^16–18^. Recent studies have hypothesized the potential formation of HClO_3_ and HClO_4_ in the lower atmosphere through observations of significant chlorate (ClO_3_^−^) and perchlorate (ClO_4_^−^) levels in rainwater, snow, and Arctic ice core samples^19–22^. Therefore, the atmospheric occurrence of chlorine oxyacids could enhance the chlorine sink, thereby affecting the oxidation capacity of the atmosphere and potentially posing environmental threats once deposited to the Earth’s surface. However, to date, there exists no direct evidence of the presence of HClO_3_ and HClO_4_ in the atmosphere, thus, limiting our full understanding of the atmospheric chlorine cycle and its associated environmental impacts.

Here, we present ambient observations of HClO_3_ and HClO_4_ in the atmosphere. Measurements were made via mass spectrometry in the Arctic at the Villum Research Station, Greenland, Ny-Ålesund, Svalbard, and over the central Arctic Ocean onboard research vessel (RV) Polarstern during the Multidisciplinary drifting Observatory for the Study of the Arctic Climate (MOSAiC) expedition. The measurements show that both chlorine oxyacids are ubiquitous and widespread during spring in the Arctic region. We find that these atmospheric species are not photoactive and therefore represent a previously unconsidered atmospheric sink of reactive chlorine in the pan-Arctic boundary layer.

## Results and discussion

### Observations of gas-phase HClO_3_ and HClO_4_ in the Arctic

**Fig. 1:**
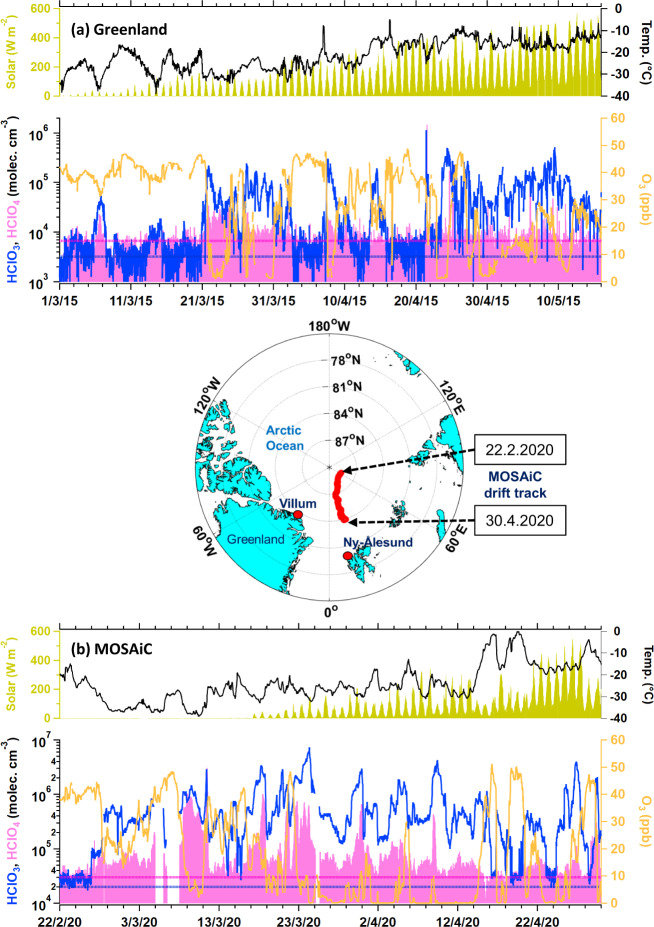
HClO_3_ and HClO_4_ over the Arctic. Time series of HClO_3_, HClO_4_, and O_3_, together with the temperature and incoming solar radiation measured at **a** the Villum Research Station from 1 March–15 May 2015 and **b** during the MOSAiC expedition from 22 February–30 April 2020. The data are displayed at a 30-min-average resolution, and any gaps in the time series are the results of instrumentation offline and maintenance periods. The dashed line represents the detection limits for HClO_3_ (blue) and HClO_4_ (pink) measurements. The uncertainty of HClO_3_ and HClO_4_ measurements was estimated to be at least a factor of two (see Methods). The map shows the location of the Villum Research Station (Nord) in Greenland, Ny-Ålesund in Svalbard, and RV Polarstern passive drifting track across the Arctic Ocean during the springtime sampling period. Note that all the time reported here is in Coordinated Universal Time (UTC). The map was created by the authors using MathWorks MATLAB (https://www.mathworks.com/products/matlab.html).

Figure 1 shows the time series of HClO_3_ and HClO_4_ measured at the Villum Research Station, Greenland, and during the MOSAiC campaign. Our observations in Greenland indicated a significant increase in the HClO_3_ signal measured with a nitrate-chemical ionization atmospheric pressure interface time-of-flight mass spectrometry (CI-APi-TOF; Methods), with an estimated concentration up to 1 × 10^6^ molecules cm^−3^ in the spring of 2015. The HClO_3_ concentration began to increase when sunlight increased towards the end of February. HClO_3_ exhibited no diurnal pattern, but a unique feature is that a significant increase in HClO_3_ concentration was observed in coincidence with the depletion of O_3_, as shown in Fig. 1a. Typically, HClO_3_ peaked under relatively low O_3_ levels (<30 ppb). We also measured HClO_3_ with a nitrate CI-APi-TOF instrument during the MOSAiC expedition in 2019/2020 (Methods section). Similar to the observation in Greenland, the measurements onboard RV Polarstern in different seasons revealed a clear increment in HClO_3_ starting at the end of February, when solar radiation started to increase after the polar night. The estimated springtime concentration of HClO_3_ during the MOSAiC campaign ranged from approximately 1 × 10^5^ to 7 × 10^6^ molecules cm^−3^ (Fig. 1b). An increase in HClO_3_ was also observed in coincidence with the depletion of O_3_ over the Arctic Ocean during the MOSAiC campaign. The HClO_3_ levels are relatively low in the other seasons, with concentrations near detection limits of ≈10^4^ molecule cm^−3^ (Supplementary Fig. S1). Further measurements at Ny-Ålesund, Svalbard also indicated the presence of HClO_3_ in springtime, with concentrations up to 8 × 10^5^ molecules cm^−3^ (Supplementary Fig. S2). However, without direct measurement of O_3_ during the campaign at Svalbard, we are not able to evaluate the relationship between HClO_3_ and O_3_.

**Fig. 2:**
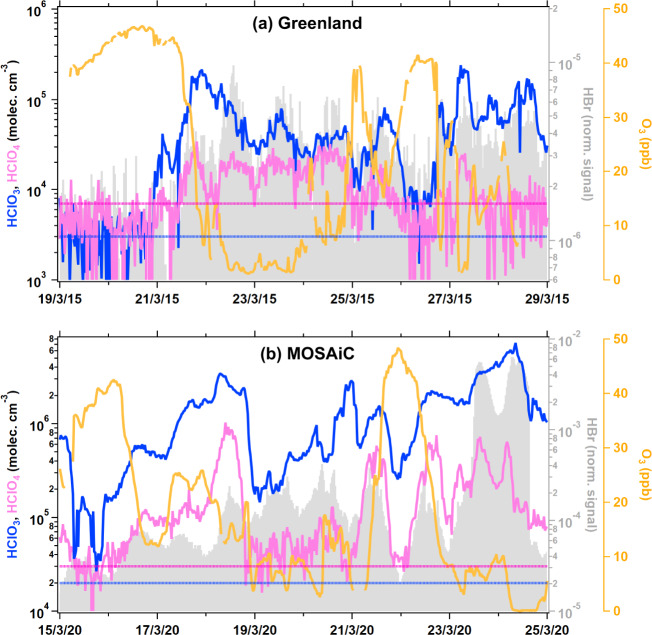
Relationships between HClO_3_, HClO_4_, O_3_, and bromine chemistry. Expanded view of HClO_3_ (blue solid line) tracking with the HClO_4_ (pink solid line) and HBr (gray shaded-area; based on the Br^–^ normalized signal from nitrate CI-APi-TOF measurements which is most likely HBr (refer to Methods) to represent bromine chemistry), at **a** the Villum Research Station, Greenland, from 19 to 29 March 2015; and **b** onboard RV Polarstern during the MOSAiC campaign, from 15 to 25 March 2020. The dashed line represents the detection limits for HClO_3_ (blue) and HClO_4_ (pink) measurements. The uncertainty of HClO_3_ and HClO_4_ measurements was estimated to be at least a factor of two.

As shown in Figs. 1, 2, the increase in HClO_3_ was accompanied by an increase in HClO_4_. The HClO_4_ concentrations in Greenland and MOSAiC were estimated to be in the range of near detection limits (7 × 10^3^) to 8 × 10^4^ molecules cm^−3^ and near detection limits (3 × 10^4^) to 1 × 10^6^ molecules cm^−3^, respectively, during springtime, which were typically lower than the HClO_3_ concentration. The lower concentration of HClO_4_ compared with that of HClO_3_ is consistent with the levels of ClO_4_^−^ and ClO_3_^−^ measured in Arctic ice cores, where the annual depositional flux of ClO_4_^−^ was reported to be several times lower than that of ClO_3_^−^ (refs. ^21,22^).

The results obtained during our campaigns at the different locations and time periods over the Arctic demonstrate that these chlorine oxyacids are widespread (Fig. 1 and Supplementary Fig. S2), and that their presence is a common phenomenon in the Arctic boundary layer during the springtime. The question arising here concerns the mechanisms leading to the occurrence of HClO_3_ and HClO_4_ in the Arctic.

### Potential formation mechanism of atmospheric chlorine oxyacids

Although the initial steps of atmospheric chlorine oxidation are well understood (1–10)^13,14^, the final oxidation steps leading to chlorine oxyacid formation are not well characterized. Here, we explore the potential formation mechanisms of the observed HClO_3_ and HClO_4_ during spring in the Arctic.

As shown in Figs. 1, 2, the increase in both HClO_3_ and HClO_4_ coincides with the decrease in the O_3_ concentration. Springtime atmospheric surface ozone destruction is a well-known phenomenon in the Arctic and is typically linked to the shallow mixing layer and chemical reactions, including bromine and chlorine chemistry^14,15,23,24^. Our air mass backward trajectory analysis revealed that chlorine oxyacid-laden and O_3_-depleted air mass originated from the near ground surface, while the high O_3_ air mass originated from higher altitudes (Supplementary Fig. S3). This indicates that the ground surface, such as snowpacks in the Arctic, may play a role in the observed increases in HClO_3_ and HClO_4_ levels. It has been suggested that the heterogeneous reaction of O_3_ on chloride-containing aqueous and salt surfaces constitutes a potential formation mechanism of ClO_3_^−^ and ClO_4_^−^ (refs. ^25–27^). However, the formation of HClO_3_ and HClO_4_ via heterogeneous reactions on the aerosol surface or direct emission from the surface of snowpacks is likely not the dominant pathway in the Arctic. This assumption is justified by the remarkably low vapor pressure (6.8 mm Hg under a 70% concentration, at 298 K)^28^ and high Henry’s law constant (*K*_H_) of HClO_4_ (9.9  ×  10^3^ mol m^−3^ Pa^−1^)^18^. Although information for *K*_H_ of HClO_3_ is not available, its value is very likely in between *K*_H_ of HClO_4_ and *K*_H_ of HOCl (6.5 mol m^−3^ Pa^−1^)^29^. These low vapor pressure and high *K*_H_ suggest that the formed ClO_3_^−^ and ClO_4_^−^ on the aerosols or snow surface are unlikely being emitted directly as gas-phase HClO_3_ or HClO_4_ into the atmosphere. This is further supported by the detection of low HClO_3_ and HClO_4_ atmospheric concentrations in winter when the Arctic is covered by snow (see Supplementary Fig. S1). Furthermore, the observed lack of a clear pattern between HClO_3_ and HClO_4_ and the aerosol surface area during the springtime (Supplementary Fig. S4) may point to their limited partitioning from the aerosol phase. Another previously suggested potential HClO_4_ formation pathway via the heterogeneous reaction of ClO with sulfuric acid (H_2_SO_4_)^30^ may also not be important, as the results demonstrated no direct relationship between HClO_3_ (or HClO_4_) and our measured H_2_SO_4_ concentrations (coefficient of determination, *R*^2^ ≤ 0.04) during both the Greenland and MOSAiC campaigns (Supplementary Fig. S4).

**Fig. 3:**
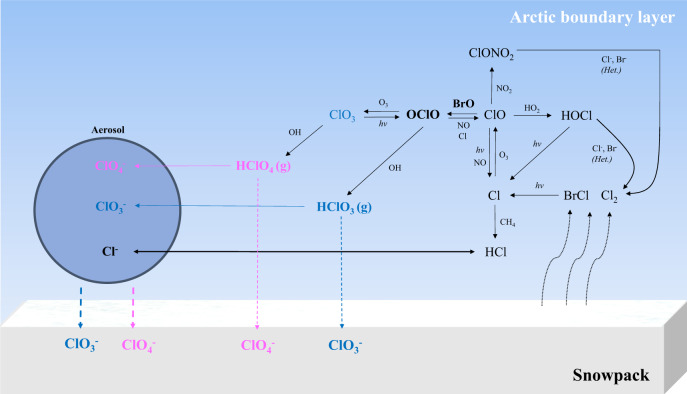
Atmospheric formation and the fate of HClO_3_ and HClO_4_. Simplified diagram of the proposed potential formation mechanism of gas-phase HClO_3_ (blue) and HClO_4_ (pink) in the Arctic boundary layer during springtime after polar sunrise. The produced HClO_3_ and HClO_4_ can be taken up by the surface of aerosols and converted into ClO_3_^−^ and ClO_4_^−^, respectively. The deposition of aerosols and/or the direct deposition of gas-phase HClO_3_ and HClO_4_ onto the ground surface, such as snowpacks, can function as a sink for reactive chlorine in the Arctic troposphere. The reactions are based on the literatures^2,12–14,64^. The mean boundary layer height was reported to vary between 100 and 200 m during the MOSAiC campaign^74,78^.

Here, we propose a more likely formation mechanism of HClO_3_ and HClO_4_ over the Arctic environment during springtime, as illustrated in Fig. 3. The snowpack emissions of Cl_2_ and BrCl^9–11^ undergo fast photolysis, leading to the production of Cl atoms, which subsequently react with O_3_ to form ClO (1–3)^3^. In addition to photolysis, the produced ClO can then react with BrO/ClO to produce OClO, or undergo loss through reactions with OH, HO_2_, NO and NO_2_, CH_3_OO, and CH_3_COOO^2^. Abundant BrO and ClO must have been present during the encountered ozone-depletion events, as have been previously demonstrated by many studies^2,3,31–37^, and significant levels of BrO have been observed in spring during the MOSAiC campaign^38^. By using the previously reported typical ranges of BrO, ClO, and HO_2_ levels during Arctic ozone-depletion events^11,32–40^, we estimate that the reaction rate of ClO + BrO is much higher than that of the ClO + ClO and ClO + HO_2_ channels (section S1 in the Supplementary Information, SI), suggesting that the increase in BrO during ozone depletion events drives the OClO formation. The reaction of ClO + OH, ClO + CH_3_OO, and ClO + CH_3_COOO are insignificant; however, the presence of typical levels of NO_x_ (NO and NO_2_) in the Arctic (i.e., 1−40 ppt) can compete with BrO for ClO (section S1 in SI). Indeed, previous ground measurements have detected significant OClO, up to 24 ppt, in the Arctic springtime^35^. Further observational evidence for the key involvement of bromine chemistry in the chlorine oxyacids formations comes from our observations which demonstrated that the recorded bromide signal adhered to the increase in HClO_3_ and HClO_4_ combined with a drastic decline in the O_3_ concentration (Fig. 2 and Supplementary Fig. S5).

OClO can undergo further reactions, including (i) reacting with Cl to yield two ClO molecules; (ii) oxidation by OH to form HClO_3_; (iii) reacting with NO to recycle ClO; and (iv) oxidation by O_3_ to produce ClO_3_ (Fig. 3). Among these reactions, OClO + OH exhibits the fastest rate, with ${k}_{{{{{{\rm{OClO}}}}}}+{{{{{\rm{OH}}}}}}}[{{{{{\rm{OH}}}}}}]/{k}_{{{{{{\rm{OClO}}}}}}+{{{{{\rm{Cl}}}}}}}[{{{{{\rm{Cl}}}}}}]$ and ${k}_{{{{{{\rm{OClO}}}}}}+{{{{{\rm{OH}}}}}}}[{{{{{\rm{OH}}}}}}]/{k}_{{{{{{\rm{OClO}}}}}}+{{{{{{\rm{O}}}}}}}_{3}}[{{{{{{\rm{O}}}}}}}_{3}]$ ratios calculated to be in the range of 2 × 10^−1^ − 1 × 10^4^ and 1 × 10^3^ − 1 × 10^6^, respectively, while the ${k}_{{{{{{\rm{OClO}}}}}}+{{{{{\rm{OH}}}}}}}[{{{{{\rm{OH}}}}}}]/{k}_{{{{{{\rm{OClO}}}}}}+{{{{{\rm{NO}}}}}}}[{{{{{\rm{NO}}}}}}]$ ratios fall in the range of 6 × 10^−1^ − 3 × 10^1^ (section S2 in the SI). These results suggest that a significant fraction of OClO can be directly oxidized by OH to convert into HClO_3_, and produce ClO to recycle OClO. This is consistent with previous experimental studies on the OH + OClO reaction, where HClO_3_ was suggested to be produced at low temperatures^41,42^. Despite the lower O_3_ concentration (a source of OH), previous studies have shown that HO_x_ (OH and HO_2_) chemistry is active during springtime in the Arctic with a reported OH concentration of ≈10^5^−10^6^ molecules cm^−3^ (refs. ^2,4,43^). This can also be indicated by our observation of significant H_2_SO_4_ concentrations (Supplementary Fig. S4), and the previously reported increase in methane sulfonic acid (MSA) levels^44,45^ during the Arctic springtime, both of which are products of sulfur oxidation reactions with OH and BrO. Therefore, OH is not a limiting factor of HClO_3_ formation in the Arctic during springtime. Given the fast reaction rate of OClO + OH, sufficient OH concentration, and enhanced OClO formation due to the increase in BrO during ozone depletion events, HClO_3_ production can occur efficiently. As to HClO_4_, the limiting factor of formation is likely the ClO_3_ concentration (Fig. 3), most likely due to its slow formation process via OClO + O_3_ (ref. ^46^). Therefore, the HClO_4_ formation is likely regulated by the O_3_ levels as indicated by the higher mean HClO_4_ concentrations observed at relatively higher O_3_ levels during the depletion events (Supplementary Fig. S6).

Based on these results, we conclude that the observed HClO_3_ and HClO_4_ over the Arctic atmosphere are predominantly produced through homogeneous reactions of chlorine, involving photochemical processes of HO_x_ and bromine chemistry.

### Atmospheric fate of HClO_3_ and HClO_4_

The fate of chlorine oxyacids determines their importance in the atmosphere. We first evaluate the potential removal of HClO_3_ and HClO_4_ in the troposphere through photodecomposition. Supplementary Fig. S7a shows our computed UV–Vis absorption spectra and cross-sections of HClO_3_ and HClO_4_ within the 170–340 nm wavelength range (refer to the Methods section for details). At the relevant wavelengths under tropospheric conditions (>290 nm), the estimated cross-sections of HClO_3_ and HClO_4_ are very small, suggesting that these two chlorine species are not photolabile in the troposphere. Based on these cross-sections, the loss rate constants of these oxyacids against photolysis at noon in the Arctic springtime are calculated as 4.4 × 10^−12^ and 2.5 × 10^−18^ s^−1^ for HClO_3_ and HClO_4_, respectively (Supplementary Fig. S7b, c).

Another possible removal pathway of HClO_3_ and HClO_4_ in the troposphere is their reactions with OH and Cl. Although there are no rate constants available for the reactions of HClO_3_ with Cl and OH, the reaction rate of HClO_3_ with either Cl or OH is expected to be low since both barriers for hydrogen abstraction from HClO_3_ are high^47^. The reactions of HClO_4_ with Cl and OH radicals (11 and 12, respectively) are also slow at low temperatures, with reported reaction rate coefficients of 1.00 × 10^−31^ and 5.8 × 10^−13^ cm^3^ molecule^−1^ s^−1^ at 253 K, respectively^48^. Assuming typical Cl (4 × 10^5^ molecules cm^−3^)^3^ and OH (5  × 10^5^ molecules cm^−3)4^ concentrations in the Arctic, the loss rates of *k*_Cl+HClO4_[Cl] and *k*_OH+HClO4_[OH] are estimated to be 4.0 × 10^−26^ and 2.9 × 10^−7^ s^−1^, respectively.

11 $${{{{\mathrm{Cl}}}}}+{{{{\mathrm{HClO}}}}}_{4}\to {{{{\mathrm{HCl}}}}}+{{{{\mathrm{ClO}}}}}_{4}$$

12 $${{{{\mathrm{OH}}}}}+{{{{\mathrm{HClO}}}}}_{4}\to {{{{\mathrm{H}}}}}_2{{{{\mathrm{O}}}}}+{{{{\mathrm{ClO}}}}}_{4}$$

Given the presence of significant aerosol particle surfaces (up to 100 µm^2^ cm^−3^) and humidity (which begins to increase in April) in the Arctic troposphere (Supplementary Fig. S8), once HClO_3_ and HClO_4_ are formed, they can undergo heterogeneous uptake on the surface of aerosol particles. Although there is no direct information available on the heterogeneous uptake coefficient (γ) of HClO_3_ and HClO_4_, previous studies have reported that the heterogeneous uptake coefficient of other chlorine acids, such as HCl, on aqueous aerosols reaches ≈0.2 at 273 K^49,50^. Both HClO_3_ and HClO_4_ are very strong acids with high electronegativity and can thus be easily ionized into ClO_3_^−^, ClO_4_^−^, and H_3_O^+^ in liquid water of the aerosol since HClO_3_ and HClO_4_ are highly soluble in water. The *K*_H_ value of HClO_4_ in water was reported as 9.9 × 10^3^ mol m^−3^ Pa^−1^ (ref. ^18^), while the *K*_H_ value of HClO_3_ likely varies between the *K*_H_ values of HClO_4_ and HOCl, with that of the latter compound reaching 6.5 mol m^−3^ Pa^−1^ (ref. ^29^). These higher *K*_H_ values than that of HCl (*K*_H_ = 0.2 mol m^−3^ Pa^−1^) may indicate that HClO_3_ and HClO_4_ could be efficiently accommodated on the surface of aerosol particles and that the fraction evaporating back into the gas phase could be small as well. By assuming that the heterogeneous uptake is accommodation limited and the γ values of HClO_3_ and HClO_4_ are similar to that of HCl (γ = 0.2)^49,50^, the estimated heterogeneous loss rate coefficients of HClO_3_ and HClO_4_ based on a typical aerosol surface area of 20 µm^2^ cm^−3^ during the MOSAiC campaign are 2.7 × 10^−4^ and 2.5 × 10^−4^ s^−1^, respectively (section S3 in SI). These rates are much (>3 orders of magnitude) higher than the rates of photodecomposition and radical attack (by OH and Cl) estimated above (<3 × 10^−7^ s^−1^). Therefore, the most relevant fate of HClO_3_ and HClO_4_ is their heterogeneous uptake by the surface of aerosol particles and subsequent deposition on the ground surface or undergo wet deposition. However, we cannot exclude the possibility of direct loss of these chlorine oxyacids to the snow surface (Fig. 3). In fact, our hypothesis is supported by previous studies in polar regions that have measured a considerable amount of ClO_3_^−^ and ClO_4_^−^ in ice cores^21,22^, snow^51^, and aerosols^52^, where atmospheric sources are strongly implicated.

Atmospheric chlorine chemistry has been regarded as a “never-ending” reaction since there is no termination process in the cycle. Indeed, the formation of HCl can serve as a sink for chlorine compounds in the troposphere, where HCl is taken up by aerosols and converted into Cl^−^, followed by an atmospheric deposition process^14,53^; although, in the presence of NO_x_ and reactive halogens (i.e., HOI and HOBr), Cl^−^ can be efficiently activated into reactive gas-phase chlorine again^54–56^. However, as HClO_3_ and HClO_4_ are not susceptible to photolysis and radical attack, and their conversion into ClO_3_^−^ and ClO_4_^−^ on aerosol surfaces or snowpacks is efficient in the Arctic boundary layer, the homogeneous formation of HClO_3_ and HClO_4_ could terminate chlorine recycling. Therefore, the formation of HClO_3_ and HClO_4_ is expected to affect the chlorine-mediated oxidation capacity in the Arctic troposphere. Furthermore, once HClO_3_ and HClO_4_ deposit on the ground surface (i.e., snowpack and sea ice), they may have environmental implications as their ions, ClO_3_^−^ and ClO_4_^−^, can accumulate in the polar ice and marine sediment, and may present a toxicity risk to resident biota^57,58^.

In summary, our study revealed the observations of HClO_3_ and HClO_4_ in the atmosphere and their widespread occurrence over the pan-Arctic during spring. We propose a novel plausible mechanism for the formation and loss of chlorine oxyacids in the Arctic environment. The results provide evidence for chlorine oxyacids to be a previously unconsidered atmospheric sink for reactive chlorine, thereby providing further insights into chlorine chemistry in the Arctic region. We, therefore, conclude that the existence of HClO_3_ and HClO_4_ in the atmosphere should be considered when evaluating the environmental impacts of chlorine chemistry in the Arctic.

## Methods

### Sampling locations

This study comprises data obtained during three field measurement campaigns over the Arctic within different time ranges. We conducted a measurement campaign at the Villum Research Station (Station Nord) in high Arctic Northern Greenland (81° 36’ N 16° 39’ W). This campaign started in mid-February 2015 and continued until the end of August 2015. The measurement station is located approximately 2 km from Station Nord, and the instrumentation was set up at the station location. We also conducted measurements at the atmospheric observatory, Gruvebadet, located 2 km southeast of Ny-Ålesund, Svalbard (78° 55’ N, 11° 56’ E), from 28 March to 30 May 2017 (spring). Detailed information on the Greenland and Ny-Ålesund sampling site can be found in ref. ^44^. The other field study was the MOSAiC expedition, which involved RV Polarstern drifting across the central Arctic from September 2019 to October 2020. The MOSAiC expedition track and detailed campaign information can be found in ref. ^59^.

### Detection of HClO_3_ and HClO_4_

A state-of-the-art chemical ionization atmospheric pressure interface time-of-flight mass spectrometry (CI-APi-TOF) instrument^60^ was employed in negative ion mode with nitrate (NO_3_^−^) ions as reagent ions to detect gas-phase HClO_3_ and HClO_4_. In Greenland, a straight, stainless steel inlet tube with a length of 1 m and an outer diameter of ¾ inch was applied at ~1.5 m above ground level (a.g.l.) to sample ambient air with a flow rate of 10 liters per minute (lpm). The inlet tube was heated to zero degrees Celsius. At Ny-Ålesund, the inlet tube length was 2 m (outer diameter of ¾ inch) and the sample was taken through the roof (height = 2 m a.g.l.), with a flow rate of 10 lpm. On RV Polarstern, the nitrate CI-APi-TOF instrument was set up in a *Swiss* Container on the bow deck of vessel^61^. The nitrate CI-APi-TOF inlet was connected to a new particle formation (NPF) inlet, through a core sampling flange system, accommodating a neutral air ion spectrometer utilized to create ~60 lpm inlet flow (height = about 15 m above sea level). The zero measurements were conducted occasionally with a high-efficiency particulate air (HEPA) filter for at least 40 min each measurement in both Greenland and MOSAiC, which cover different seasons during the measurement period.

HClO_3_ was detected as ClO_3_^−^ (82.954 *m/z*), and its isotope peak was clearly observed at 84.951 *m/z*, with a ^35^Cl:^37^Cl ratio of approximately 3:1 (Supplementary Fig. S9). Regarding HClO_4_ detection, the peak of ClO_4_^−^ (98.949 *m/z*) should be carefully identified, as it is difficult to distinguish it from the isotopic peak of deprotonated sulfuric acid, HSO_4_^−^ (i.e., H^34^SO_4_^−^ = 98.956 *m/z*) when the HSO_4_^−^ signal is high. The *m/z* values of these two peaks are close to each other and may create interference with lower-mass resolution devices. Therefore, we considered the peak of ^37^ClO_4_^−^ (100.946 *m/z*) to estimate the HClO_4_ signal in this study. We also detected the Br^−^ signal from the peak at 78.919 *m/z* (together with NO_3_(HBr)^−^ peak at 141.915 *m/z*; Supplementary Fig. S10), which is most likely attributed to hydrobromic acid (HBr)^62^. The raw data was pre-averaged over 10 min and processed with the MATLAB tofTools package according to the procedures described in Jokinen et al.^60^.

The detected ClO_3_^−^ and ClO_4_^−^ were the deprotonated products of HClO_3_ and HClO_4_, respectively. Quantum chemical calculations (section S4 in SI) indicated that the binding free energies of NO_3_^−^ with HClO_3_ and HClO_4_ are 24.0 and 36.9 kcal mol^−1^, respectively (the binding free energy is simply −1 × formation free energy in Supplementary Fig. S11), and the deprotonation free energies are 7.1 and 24.8 kcal mol^−1^, respectively, which suggests that both HClO_3_ and HClO_4_ are efficiently deprotonated into ClO_3_^−^ and ClO_4_^−^, respectively. The HClO_3_•NO_3_^−^ cluster remains more stable against deprotonation than the HClO_4_•NO_3_^−^ cluster, and some fraction of the former could occur, which is consistent with the detection of the HClO_3_•NO_3_^−^ cluster (refer to Supplementary Fig. S9c,d), while the HClO_4_•NO_3_^−^ cluster was not present in the spectrum. The isomers of HClO_3_ and HClO_4_ detected via nitrate CI-APi-TOF likely occurred in the form of HOClO_2_ and HOClO_3_, respectively, as these components are most energetically stable in the atmosphere^42,63,64^.

Currently, there are no available methods for HClO_3_ and HClO_4_ calibration of CI-APi-TOF instruments. More importantly, the handling of HClO_3_ and HClO_4_ is dangerous, as these substances are very corrosive and could cause violent explosions in reactions with organics, making calibration becoming very difficult. Based on quantum chemical calculations, the binding free energies of NO_3_^−^ with HClO_3_ and HClO_4_, i.e., ${{{{{\mathrm{HClO}}}}}}_{3} \bullet {{{{{{\mathrm{NO}}}}}}_{3}^{-}}\to {{{{{{\mathrm{NO}}}}}}_{3}^{-}}+{{{{{\mathrm{HClO}}}}}}_{3}$ and ${{{{{\mathrm{HClO}}}}}}_{4}\bullet {{{{{{\mathrm{NO}}}}}}_{3}^{-}}\to {{{{{{\mathrm{NO}}}}}}_{3}^{-}}+{{{{{\mathrm{HClO}}}}}}_{4}$ (24.0 and 36.9 kcal mol^−1^, respectively), are similar to (the former is slightly lower than) that of ${{{{{{\mathrm{NO}}}}}}_{3}^{-}}$ with H_2_SO_4_ (${{{{{\mathrm{H}}}}}}_{2}{{{{{\mathrm{SO}}}}}}_{4}\bullet {{{{{{\mathrm{NO}}}}}}_{3}^{-}}\to {{{{{{\mathrm{NO}}}}}}_{3}^{-}}+{{{{{\mathrm{H}}}}}}_{2}{{{{{\mathrm{SO}}}}}}_{4}$; 34.4 kcal mol^−1^). However, similar to H_2_SO_4_ (${{{{{\mathrm{H}}}}}}_{2}{{{{{\mathrm{SO}}}}}}_{4}\bullet {{{{{{\mathrm{NO}}}}}}_{3}^{-}}\to {{{{{{\mathrm{HSO}}}}}}_{4}^{-}}+{{{{{\mathrm{HNO}}}}}}_{3}$ = 20.3 kcal mol^−1^), their binding free energies are higher than their deprotonation free energies (7.1 and 24.8 kcal mol^−1^ for ${{{{{\mathrm{HClO}}}}}}_{3}\bullet {{{{{{\mathrm{NO}}}}}}_{3}^{-}}\to {{{{{{\mathrm{ClO}}}}}}_{3}^{-}}+{{{{{\mathrm{HNO}}}}}}_{3}$ and ${{{{{\mathrm{HClO}}}}}}_{4}\bullet {{{{{{\mathrm{NO}}}}}}_{3}^{-}}\to {{{{{{\mathrm{ClO}}}}}}_{4}^{-}}+{{{{{\mathrm{HNO}}}}}}_{3}$, respectively), which suggests that deprotonation of the formed clusters is efficient. Therefore, if HClO_3_ and HClO_4_ are dissociated during ionization, they could preferably form ClO_3_^−^ and ClO_4_^−-^, respectively, and are detectable via nitrate CI-APi-TOF. This indicates that the detection of HClO_3_ and HClO_4_ is very likely as efficient as the detection of H_2_SO_4_ by NO_3_^−^, whose reaction rate is expected to occur within the kinetic limit range^65^. Thus, it is reasonable to assume that the instrument sensitivity to HClO_3_ and HClO_4_ is similar to the sensitivity determined for the H_2_SO_4_ measurement.

H_2_SO_4_ calibration for the nitrate CI-APi-TOF was conducted before or immediately after the campaign with a method presented in ref. ^66^. Regarding the Greenland and Ny-Ålesund measurements, the obtained H_2_SO_4_ calibration factor was 1.48 × 10^9^ (ref. ^44^). During the MOSAiC campaign, calibration was completed twice after the campaign, and the average of two-factor values was 6 × 10^9^. This factor includes the losses at the NPF inlet. Based on these calibration factors, the detection limits of HClO_3_ and HClO_4_ were estimated as 3 × 10^3^ and 7 × 10^3^ molecules cm^−3^ (10 min-average, 3*σ*), respectively, during the Greenland measurement campaign and 2 × 10^4^ and 3 × 10^4^ molecules cm^−3^ (10 min-average, 3*σ*), respectively, during the MOSAiC measurement campaign. The detection limit for HClO_3_ measurements in Ny-Ålesund was calculated to be 1 × 10^3^ molecules cm^−3^ (10 min-average, 3*σ*).

The HClO_3_ and HClO_4_ concentrations in this study were computed with Eq. (13).

13 $$\left[{{{{{\mathrm{HClO}}}}}}_{{{{{\mathrm{x}}}}}}\right]=C{{\times }}\frac{{{{{{{\mathrm{ClO}}}}}}_{{{{{{\mathrm{x}}}}}}}}^{-}+({{{{{\mathrm{HClO}}}}}}_{{{{{\mathrm{x}}}}}})\bullet {{{{{{\mathrm{NO}}}}}}_{{3}}}^{-}}{{{{{{{\mathrm{NO}}}}}}_{{3}}}^{-}+\left({{{{{\mathrm{HNO}}}}}}_{3}\right)\bullet {{{{{{\mathrm{NO}}}}}}_{{3}}}^{-}}$$

where x equals 3 or 4, and *C* is the calibration factor, which was assumed to be similar to the H_2_SO_4_ calibration factor. If the detected HClO_3_ and HClO_4_ clusters are not charged as efficiently as H_2_SO_4_, it could lead to underestimating the concentration of HClO_3_ and HClO_4_. The sum uncertainties of the HClO_3_ and HClO_4_ measurement from the collision limit of the target compound with its charger ions and potential inlet losses were predicted to be at least a factor of two.

### Ancillary measurements

O_3_ was measured with a UV ozone analyser during both the Greenland and MOSAiC campaigns. More details on the O_3_ measurement setup in Greenland can be found in ref. ^67^. Regarding the MOSAiC O_3_ measurements, we used here an hourly merged dataset that combines cross-evaluated measurements performed by three independent instruments, as has been detailed in refs. ^68,69^. The particle number size distribution, 10–500 nm (9–915 nm in Greenland), was measured with a scanning mobility particle sizer (SMPS). The information on the SMPS setup in Greenland can be found in ref. ^70^. As for the MOSAiC campaign, the SMPS was measured from the United States Department of Energy Atmospheric Radiation Measurement (ARM) Aerosol Observation System container^71,72^. Water vapor was measured during the MOSAiC campaign via cavity ring-down spectroscopy (CRDS) with a commercial Picarro instrument (model G2401) also connected to the interstitial inlet of the Swiss container. Meteorological parameters (temperature and solar radiation) during the MOSAiC were obtained from the meteorological observatory Polarstern^73^. Details on the atmospheric and meteorological equipment during the MOSAiC campaign can be found in ref. ^59^ and ref. ^74^.

### HClO_3_ and HClO_4_ photolysis rates

To obtain the photolysis rates of HClO_3_ and HClO_4_, we applied the estimated absorption cross-sections of these two compounds (refer to section S5 in SI for the cross-sections computation) in an explicit Tropospheric Halogen Chemistry Model (THAMO): a one-dimensional atmospheric chemistry model^75^ that has been used in many previous studies (e.g., ref. ^56^) to simulate the halogen chemical processes (including the photolysis) in the boundary layer. The THAMO simulations were conducted for 24 h in an Arctic environment (with a latitude of 81° 21′ N, similar to the location of Villum Research Station, Greenland) during spring (1 May) to derive the photolysis rates of HClO_3_ and HClO_4_ in the Arctic boundary layer.

## Supplementary information

Supplementary Information

Peer Review File

## Data Availability

The data that support the findings of this study are available online in the following repositories. All of the data obtained from Greenland (Villum Research Station) and Ny-Ålesund observations, and absorption cross-sections and photolysis rates of HClO_3_ and HClO_4_ have been deposited in Zenodo: 10.5281/zenodo.7655981 and 10.5281/zenodo.4292239 (for H_2_SO_4_). The MOSAiC dataset used in this study are available at 10.1594/PANGAEA.944393 (for merged O_3_), https://doi.pangaea.de/10.1594/PANGAEA.956085 (for HClO_3_ and HClO_4_), https://doi.pangaea.de/10.1594/PANGAEA.956087 (for HBr and H_2_SO_4_), 10.5439/1225453 (Kuang et al.^72^ for the particle number size distribution), 10.1594/PANGAEA.935265 (for meteorological data), 10.1594/PANGAEA.954232 (for water vapor), and 10.17632/bn7ytz4mfz.1 (for BrO).
