# Supplementary material for: Widespread detection of chlorine oxyacids in the Arctic atmosphere
Source: Nat Commun. 2023 Mar 30;14:1769. doi: 10.1038/s41467-023-37387-y (PMC10063661; doi:10.1038/s41467-023-37387-y)
Supplement: Supplementary file 1 — Supplementary Information [file 41467_2023_37387_MOESM1_ESM.pdf]

Supplementary Information

Widespread detection of chlorine oxyacids in the Arctic atmosphere

Yee Jun Tham[1,2,3]*, Nina Sarnela[1], Siddharth Iyer[4], Qinyi Li[5,19], Hélène Angot[6,7], Lauriane L. J. Quéléver[1], Ivo Beck[6], Tiia Laurila[1], Lisa J. Beck[1], Matthew Boyer[1], Javier Carmona-García[8], Ana Borrego-Sánchez[9], Daniel Roca-Sanjuán[8], Otso Peräkylä[1], Roseline C. Thakur[1], Xu-Cheng He[1], Qiaozhi Zha[1], Dean Howard[10,11,12], Byron Blomquist[11,12], Stephen D. Archer[13], Ludovic Bariteau[11.12], Kevin Posman[13], Jacques Hueber[10,20], Detlev Helmig[10,21], Hans-Werner Jacobi[7], Heikki Junninen[14], Markku Kulmala[1], Anoop S. Mahajan[15], Andreas Massling[16], Henrik Skov[16], Mikko Sipilä[1], Joseph S. Francisco[17], Julia Schmale[6], Tuija Jokinen[1,18]* and Alfonso Saiz-Lopez[5]*

[1] Institute for Atmospheric and Earth System Research/Physics, Faculty of Science, University of Helsinki, 00014 Helsinki, Finland;
[2] School of Marine Sciences, Sun Yat-sen University, Zhuhai 519082, China;
[3] Guangdong Provincial Key Laboratory of Marine Resources and Coastal Engineering, Zhuhai, 519082, China;
[4] Aerosol Physics Laboratory, Tampere University, Tampere, FI-33720, Finland;
[5] Department of Atmospheric Chemistry and Climate, Institute of Physical Chemistry Rocasolano, CSIC, Madrid 28006, Spain;
[6] Extreme Environments Research Laboratory, École Polytechnique Fédérale de Lausanne, (EPFL) Valais Wallis, Sion, Switzerland;
[7] Univ. Grenoble Alpes, CNRS, INRAE, IRD, Grenoble INP, IGE, 38000 Grenoble, France;
[8] Institut de Ciència Molecular, Universitat de València, P.O. Box 22085, València 46071, Spain;
[9] Instituto Andaluz de Ciencias de la Tierra, CSIC-University of Granada, Av. de las Palmeras 4, 18100 Armilla, Granada, Spain;
[10] Institute of Arctic and Alpine Research, University of Colorado, Boulder, CO, 80309, USA;
[11] Cooperative Institute for Research in Environmental Science, University of Colorado, Boulder, CO, 80309, USA;
[12] Physical Sciences Laboratory, National Oceanic and Atmospheric Administration, Boulder, CO, 80305, USA;
[13] Bigelow Laboratory for Ocean Sciences, East Boothbay, Maine, USA;
[14] Laboratory of Environmental Physics, Institute of Physics, University of Tartu, Tartu, Estonia;
[15] Indian Institute of Tropical Meteorology, Ministry of Earth Sciences, Pune, 411008, India;
[16] Department of Environmental Science, iClimate, Aarhus University, Roskilde, Denmark;
[17] Department of Earth and Environmental Sciences and Department of Chemistry, University of Pennsylvania, Philadelphia, Pennsylvania 19104, USA;
[18] Climate and Atmosphere Research Centre (CARE-C), the Cyprus Institute, P.O. Box 27456, Nicosia, CY-1645, Cyprus.
[19] Present address: Department of Civil and Environmental Engineering, The Hong Kong Polytechnic University, Hong Kong, China;
[20] Present address: JH Atmospheric Instrumentation Design, Boulder, CO, USA;
[21] Present address: Boulder Atmosphere Innovation Research LLC, Boulder, CO, USA;

***Corresponding authors**: Yee Jun Tham (thamyj@mail.sysu.edu.cn); Tuija Jokinen (t.jokinen@cyi.ac.cy); Alfonso Saiz-Lopez (a.saiz@csic.es)

Text

S1. Calculation of gas-phase OClO production rate. The production of OClO is a key step in the gas-phase formation of $HClO_3$ and $HClO_4$, and ClO is an important precursor of OClO. As shown in Fig.3, apart from the photolysis, the ClO can react with BrO and ClO to form OClO, with HO_2 to produce HOCl, and with NO_x (NO and NO_2) to form Cl and $ClONO_2$, respectively. To evaluate the dominance of ClO reaction pathways, we calculate the reaction rate of ClO with BrO, ClO, HO_2, NO and NO_2 as in reactions (S1) to (S5). The reaction rate coefficient (k) is adopted from the IUPAC

kinetic database[1] with the reaction temperature set at 253 K, which is a typical Arctic temperature during spring (refer to Fig. 1).

$$ClO + BrO \overset{k_{S1}}{\rightarrow} OClO + Br \qquad k_{S1} = 8.75 \times 10^{-12}\ cm^3\ molecule^{-1}\ s^{-1} \qquad (S1)$$

$$ClO + ClO \overset{k_{S2}}{\rightarrow} OClO + Cl \qquad k_{S2} = 1.56 \times 10^{-15}\ cm^3\ molecule^{-1}\ s^{-1} \qquad (S2)$$

$$ClO + HO_2 \overset{k_{S3}}{\rightarrow} HOCl + O_2 \qquad k_{S3} = 8.43 \times 10^{-12}\ cm^3\ molecule^{-1}\ s^{-1} \qquad (S3)$$

$$ClO + NO \overset{k_{S4}}{\rightarrow} Cl + NO_2 \qquad k_{S4} = 2.04 \times 10^{-11}\ cm^3\ molecule^{-1}\ s^{-1} \qquad (S4)$$

$$ClO + NO_2 \overset{k_{S5}}{\rightarrow} ClONO_2 \qquad k_{S5} = 7.10 \times 10^{-12}\ cm^3\ molecule^{-1}\ s^{-1} \qquad (S5)$$

In this calculation, we used the BrO, ClO and HO_2 concentrations that have been previously observed in the Arctic during spring. Typically, the BrO concentrations were reported to peak in the range of about 1.5−10.2 × 10^8 molecules cm^{-3} (5−35 parts per trillion, ppt) during an Arctic ozone depletion event[2-6] and MOSAiC campaign[7]. The daytime maximum ClO concentration was reported to be in the range of 1.5−11.6 × 10^8 molecules cm^{-3} (5−40 ppt) in previous field observations in the Arctic springtime[5,8,9]. The HO_2 concentration was typically measured to peak at approximately 1.5−2.9 × 10^8 molecules cm^{-3} (5−10 ppt) in the springtime O_3 depletion events[10,11]. NO_x data were obtained from the Atmospheric Tomography (ATom) aircraft measurements over the Arctic (70–90°N) marine boundary layer (altitude <1 km), using the National Oceanic and Atmospheric Administration (NOAA) chemiluminescence instrument described in Bourgeois *et al.*[12]. The NO and NO_2 concentrations ranging from 0.3−4.4 × 10^8 molecules cm^{-3} (1−15 ppt) and 0.6−11.6 × 10^8 molecules cm^{-3} (2−40 ppt), respectively[13].

Supplementary Fig. S12 shows the range of ClO loss rates calculated with the above concentration ranges of BrO, ClO, HO_2, NO and NO_2. The ClO + BrO reaction channel is the dominant (rates up to 1.0 × 10^7 molecules cm^{-3} s^{-1}), followed by the the ClO + HO_2 (rates up to 2.9 × 10^6 molecules cm^{-3} s^{-1}). The reaction of ClO + ClO is negligible as the rate (up to 2.1 × 10^3 molecules cm^{-3} s^{-1}) is several orders of magnitude lower than the former two reaction channels. Although the k_{S1} and k_{S3} are quite comparable, the BrO concentration during an Arctic ozone depletion event is usually several times higher than HO_2 concentrations[8,11], suggesting that the ClO + BrO channel can lead to the enhancement of OClO, and contribute to the formation of $HClO_3$ and $HClO_4$. Furthermore, the fraction of ClO reacting with HO_2 to form HOCl can recycle to produce Cl atoms via direct photolysis or heterogeneous uptake on chloride aerosol, which can eventually form ClO. The calculated reaction rates of ClO + NO (up to 1.0 × 10^7 molecules cm^{-3} s^{-1}) and ClO + NO_2 (up to 9.6 × 10^6 molecules cm^{-3} s^{-1}) are comparable to the reaction rate of ClO + BrO (Fig. S3b), meaning that the typical levels of NO_x will compete with BrO in the reaction of ClO to form OClO.

According to the Thompson *et al.*[8], the ClO can also react with radicals such as OH, CH_3OO, and CH_3COOO. However, the reactions of ClO with OH, CH_3OO and CH_3COOO are very likely not important compared to the losses of ClO through HO_2 and BrO for two reasons. First, according to the reaction rate constants used in Thompson *et al.*[8], the ClO + CH_3OO (2.08 × 10^{-12} cm^3 $molecule^{-1}$ s^{-1}) and ClO + CH_3COOO (2.03 × 10^{-12} cm^3 $molecule^{-1}$ s^{-1}) are about 4 times slower than the ClO + HO_2 (8.67 × 10^{-12} cm^3 $molecule^{-1}$ s^{-1}), while the reaction constant for ClO + OH (2.37 × 10^{-13} cm^3 $molecule^{-1}$ s^{-1}) is an order of magnitude slower than the reaction constant for ClO + HO_2. Second, the concentrations of CH_3OO and CH_3COOO in the Arctic are likely smaller than concentration of HO_2 because their lifetime may be short as these RO_2 radicals typically react more rapidly with NO or HO_2[8]. Even if we assume that their concentration equals to the HO_2 concentration (i.e. 2.9×10^8 molecules cm^{-3}), the loss rates of ClO (i.e., at a concentration of 5.8 × 10^8 molecules cm^{-3}) by reacting with CH_3OO and CH_3COOO are 3.5 × 10^5 and 3.4 × 10^5 molecules cm^{-3} s^{-1}, respectively, which are much lower than the ClO + HO_2 pathway (refer to Supplementary Fig. S12). The loss rate of ClO (i.e., at a concentration of 5.8 × 10^8 molecules cm^{-3}) by reacting with 5 ×10^6 molecules cm^{-3} of OH is negligible (rate is 6.9 × 10^2 molecules cm^{-3} s^{-1}).

S2. Estimating the loss of OClO. The loss of OClO is another important intermediate step for $HClO_3$ and $HClO_4$ formation. Here, we calculate the consumption rate of OClO by OH, Cl, O_3 and NO, as shown in the reactions (S6) to (S9). The reaction rate coefficient (*k*) is adopted from the IUPAC kinetic database[1] and Zhu and Lin study[14], with the reaction temperature set at 253 K.

$$OClO + OH \xrightarrow{k_{S6}} HClO_3 \qquad k_{S6} = 1.32 \times 10^{-10}\ cm^3\ molecule^{-1}\ s^{-1} \qquad (S6)$$

$$OClO + Cl \xrightarrow{k_{S7}} 2ClO \qquad k_{S7} = 6.27 \times 10^{-11}\ cm^3\ molecule^{-1}\ s^{-1} \qquad (S7)$$

$$OClO + O_3 \xrightarrow{k_{S8}} ClO_3 + O_2 \qquad k_{S8} = 1.79 \times 10^{-20}\ cm^3\ molecule^{-1}\ s^{-1} \qquad (S8)$$

$$OClO + NO \xrightarrow{k_{S9}} ClO + H_2O \qquad k_{S9} = 1.51 \times 10^{-13}\ cm^3\ molecule^{-1}\ s^{-1} \qquad (S9)$$

Supplementary Table S1 shows the estimated consumption rate of OClO by OH, Cl, O_3 and NO. When OH concentration is about 0.1−1.0 × 10^6 molecules cm^{-3}, a typical range of OH during O_3 depletion as reported in previous Arctic measurements[8,15,16], the $k_{\mathrm{OClO+OH}}[\mathrm{OH}]$ is ≈10^{-4}−10^{-5} s^{-1}. Cl atom was reported to be in the range of ≈10^3−10^6 molecules cm^{-3} in Arctic spring[5,9,15,17]. The $k_{\mathrm{OClO+Cl}}[\mathrm{Cl}]$ is calculated to be ≈ 6 × 10^{-5}−10^{-8} s^{-1}. From Fig.1, the typical O_3 concentration during an O_3 depletion event falls within the range of 3 × 10^8−6 × 10^{11} molecule cm^{-3} (1−20 parts per billion, ppb), the $k_{\mathrm{OClO+O3}}[O_3]$ are estimated to be in between ≈ 1 × 10^{-8}−5 × 10^{-8} s^{-1}. From the ATom aircraft measurements over the Arctic in May 2018[13], the mean concentration was 1.5×10^8 molecules cm^{-3} (5 ppt) and the maximum concentration was 4.4×10^8 molecules cm^{-3} (15 ppt). When NO concentration is 4.4 × 10^8 molecules cm^{-3}, $k_{\mathrm{OClO+NO}}$ is 6.6 × 10^{-5} s^{-1}, while the $k_{\mathrm{OClO+NO}}$ is 2.2 × 10^{-5} s^{-1} when the NO concentration is 1.5 × 10^8 molecules cm^{-3}. Note that the reaction of OClO + NO will produce ClO, which can recycle OClO via reaction RS1. Supplementary Fig. S13 shows that there are significant levels of $HClO_3$ and $HClO_4$ during the measurement period with potential influence of ship pollution in the MOSAiC campaign, where a relatively high probably that NO_x levels were elevated during these periods. Therefore, among these reactions, OClO + OH represents a significant fraction of OClO loss (up to several orders of magnitude higher than other pathways), ultimately promoting the $HClO_3$ formation.

S3. Heterogeneous loss of $HClO_3$ and $HClO_4$ on aerosol. To estimate the potential removal of $HClO_3$ and $HClO_4$ through heterogeneous reaction with aerosol, we calculated the heterogeneous loss rate coefficient (K_{het}) of $HClO_3$ and $HClO_4$ based on the following equation (S10).

$$K_{\mathrm{het}} = \frac{1}{4} \times S \times c \times \gamma \qquad (S10)$$

Where S is the aerosol surface area, c is the molecular speed of $HOCl_3$ and $HOCl_4$, respectively, and γ is the heterogeneous uptake coefficient, which was assumed to be 0.2 (similar to the γ of HCl when assuming the uptake is accommodation limited). The c of $HOCl_3$ and $HOCl_4$ were estimated by equation (S11)[18].

$$\sqrt{\frac{8RT}{\pi(MW)}} \qquad (S11)$$

The R is the universal gas constant (8.314 J K^{-1} mol^{-1}), T is the temperature (253 K), and MW is the molecular weight of $HOCl_3$ (84.459 g mol^{-1}) and $HOCl_4$ (100.460 g mol^{-1}). Based on the typical aerosol surface area of 20 μm^2 cm^{-3} in MOSAiC, the K_{het} of $HClO_3$ and $HClO_4$ are estimated to be 2.7×10^{-4} and 2.5×10^{-4} s^{-1}, respectively. With the highest aerosol surface area of 100 μm^2 cm^{-3} observed in spring, for instance in April (see Supplementary Fig. S4 and S8), the calculated K_{het} of $HClO_3$ and $HClO_4$ are 1.4×10^{-3} and 1.3×10^{-3} s^{-1}, respectively. Note that the actual γ for both $HOCl_3$ and $HOCl_4$ are unknown, and a change in γ may affect the heterogeneous loss rate of $HOCl_3$ and $HOCl_4$. If we assume that the γ is in unity, the heterogeneous loss rate of $HOCl_3$ and $HOCl_4$ falls within 1.4−6.8 ×10^{-3} s^{-1} and 1.3−6.3 × 10^{-3} s^{-1}, respectively (Supplementary Table S2). When the γ is assumed to be equal to a smaller γ (0.01), the rates are 1.4−6.8 ×10^{-5} s^{-1} (for $HClO_3$) and 1.3−6.3 × 10^{-5} s^{-1} (for $HClO_4$). These predicted K_{het} are much higher than those rates determined from the photolysis and radical attack for $HClO_3$ and $HClO_4$. The result suggests that the heterogeneous loss is an important loss pathway for $HClO_3$ and $HOCl_4$ in the Arctic.

S4. Quantum chemical calculations of binding energies. The cluster formation free energies of $HClO_3$, $HClO_4$ and nitrate ions were calculated using quantum chemical methods. Conformers were generated using the MMFF method in the Spartan '16 program[19]. The geometries were then

optimized using density functional theory (DFT) methods at the ωB97X-D/aug-cc-pVTZ level of theory[20,21]. The DFT calculations were carried out using the Gaussian 16 program[22]. A final coupled-cluster single-point energy calculation on the lowest energy geometries was carried out with the domain-based local pair natural orbital coupled-cluster singles, doubles and perturbative triples (DLPNO-CCSD(T)) method and the def2-QZVPP basis set using the ORCA program[23,24].

S5. Computation of $HClO_3$ and $HClO_4$ absorption cross-sections. The UV-Vis electronic absorption spectrum and cross-sections of $HClO_3$ and $HClO_4$ were obtained using a well-established nuclear-ensemble approach previously applied to several systems of atmospheric interest[25-29]. This computational strategy consists firstly of the generation of a representative ensemble of structures for each system by sampling the ground-state equilibrium structure, optimized at the B3LYP/6-31G** level of theory with the Gaussian 16 (revision C0.1) program[22], according to a Wigner distribution at 0 K using the Newton-X 2.0 software package[30]. Then, for each structure, vertical excitation energies (ΔE) and oscillator strengths (f) are computed with state-average complete-active-space self-consistent field/multi-state complete-active space second-order perturbation theory/spin-orbit complete-active-space state interaction (SA-CASSCF/MS-CASPT2/SO-CASSI) scheme with the third-order Douglas-Kroll and Hess (DKH3) Hamiltonian and the atomic natural orbital-relativistic correlation consistent basis set of valence triple zeta accuracy with polarization functions (ANO-RCC-VTZP) using the OpenMolcas software package[31,32]. An active space of 12 electrons in 9 orbitals and 16 electrons in 12 orbitals was considered for $HClO_3$ and $HClO_4$, respectively. Calculations were carried out without symmetry restrictions (C_1 point group) and considering 8 and 7 roots for singlet and triplet multiplicities, respectively (8 singlet states and 7 triplet states). The ionization potential electron affinity (IPEA) parameter was set to the recommended value of 0.25 a.u to correct the errors in energy differences between closed- and open-shell systems[33], and an imaginary level shift of 0.2 a.u. was applied to minimize the effect of weakly-interacting intruder states[34]. Spin-orbit couplings (SOCs) were computed with the complete-active-space state interaction (CASSI) method, and f values were determined as described in previous works[25-29]. Finally, at each photon energy E, the corresponding absorption cross-section $\sigma(E)$ are obtained by convoluting the calculated electronic transitions using a Gaussian-type shape function centred at each vertical excitation energy, accounting for the broadening of the resonant lines of the spectra through full width at half-maximum (FWHM) set to 0.1 eV, obtaining in this way physically meaningful bandshapes without producing any unphysical peaks.

Supplementary Tables

Supplementary Table S1 | Loss of OClO. The calculated consumption rate of OClO by OH, Cl, O_3 and NO. The reaction temperature is 253 K. Concentration is in molecules cm^{-3}.

Reaction	OClO + OH		OClO + Cl		OClO + O_3		OClO + NO	
[X]	[OH] = 5×10^{6}	[OH] = 1×10^{5}	[Cl] = 1×10^{6}	[Cl] = 1×10^{3}	[O_3] = 6×10^{11}	[O_3] = 3×10^{10}	[NO] = 4.4×10^{8}	[NO] = 1.5×10^{8}
k_{OClO+X}[X] (s^{-1})	6.6×10^{-4}	1.3×10^{-5}	6.3×10^{-5}	6.3×10^{-8}	1.0×10^{-8}	5.2×10^{-10}	6.6×10^{-5}	2.2×10^{-5}

Note: X = OH; Cl; O_3 or NO

Supplementary Table S2 | Heterogeneous loss of $HClO_3$ and $HClO_4$ on aerosol. The predicted heterogeneous loss rate coefficient of $HClO_3$ and $HClO_4$ with various uptake coefficients and typical aerosol surface area in the Arctic environment. The aerosol surface area is in μm cm^{-3}.

	γ = 0.2		γ = 0.01		γ = 1	
	Surf. area = 20	Surf. area = 100	Surf. area = 20	Surf. area = 100	Surf. area = 20	Surf. area = 100
$HClO_3$ het. loss rate coefficient (s^{-1})	2.7×10^{-4}	1.4×10^{-3}	1.4×10^{-5}	6.8×10^{-5}	1.4×10^{-3}	6.8×10^{-3}
$HClO_4$ het. loss rate coefficient (s^{-1})	2.5×10^{-4}	1.3×10^{-3}	1.3×10^{-5}	6.3×10^{-5}	1.3×10^{-3}	6.3×10^{-3}

Supplementary Figures

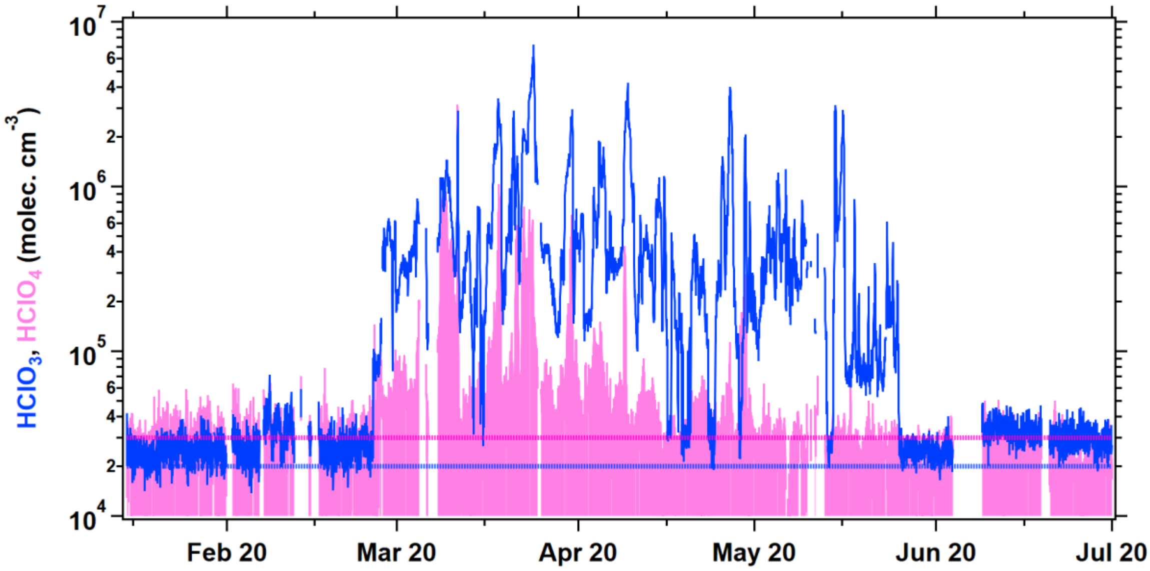

Supplementary Fig. S1 | $HClO_3$ and $HClO_4$ measurements during the MOSAiC campaign. The variation in the $HClO_3$ (blue) and $HClO_4$ (red) concentrations observed from January to June 2020 during the MOSAiC campaign. This period covers the winter, spring and summer in the Arctic. The dashed-line represents the detection limits for $HClO_3$ (blue) and $HClO_4$ (pink) measurements. The uncertainty of $HClO_3$ and $HClO_4$ measurements was estimated to be at least a factor of two (see Methods).

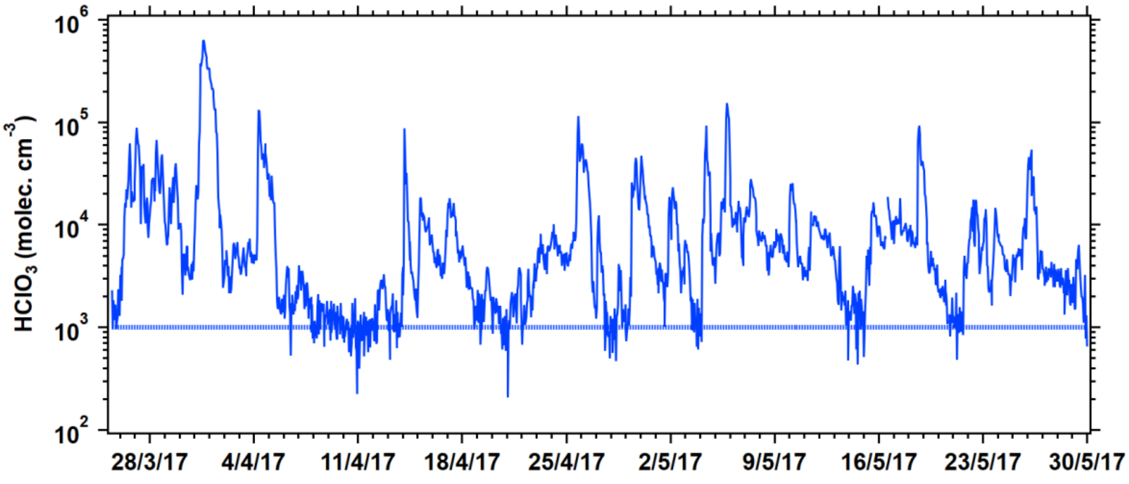

Supplementary Fig. S2 | Observation of $HClO_3$ at Ny-Ålesund. Example of the $HClO_3$ concentration data measured with a nitrate-CI-APi-TOF instrument at Ny-Ålesund, Svalbard (78° 55' N, 11° 56' E), from 28 March to 30 May 2017 (spring). The measurements occurred at the atmospheric observatory, Gruvebadet, located 2 km southeast of Ny-Ålesund (refer to the map in Fig. 1). The dashed-line represents the detection limit for $HClO_3$ measurement. The uncertainty of $HClO_3$ and $HClO_4$ measurements was estimated to be at least a factor of two (see Methods). Details on the site (description), instrumentation setup and calibration can be found in Beck *et al*.[35].

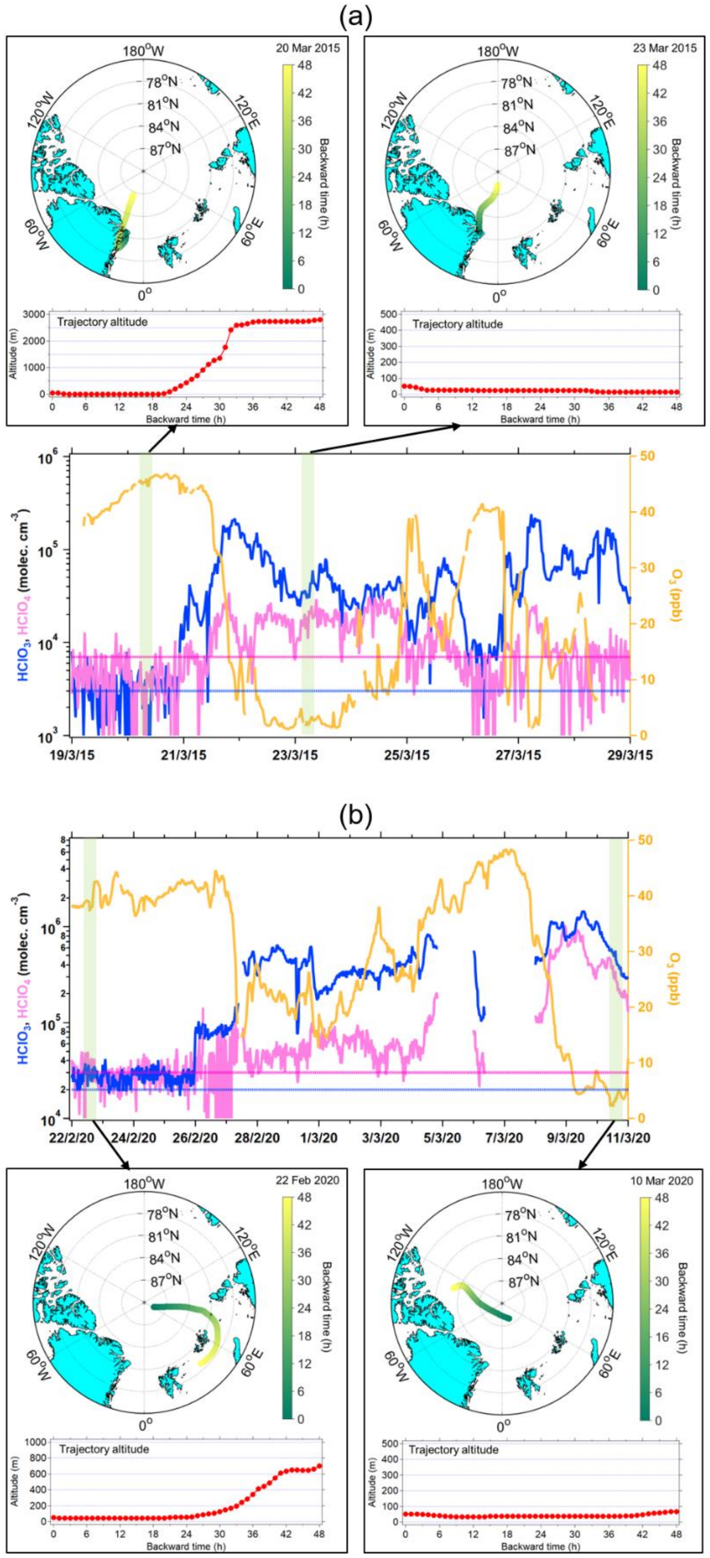

Supplementary Fig. S3 | Air mass trajectories during non-O_3 depletion and O_3 depletion events. Example cases (area shaded in light green) revealing the difference between the air mass origin and height during ozone depletion events and non-ozone depletion events at the (**a**) Villum Research Station, Greenland, and during the (**b**) MOSAiC campaign. The 48-hour backward air mass trajectory and altitude are calculated with the Hybrid Single-Particle Lagrangian Integrated Trajectory (HYSPLIT) model, developed by the NOAA Air Resources Laboratory[36]. The starting height of the trajectory was set at 50 m above ground level. Maps were created by the authors using MathWorks MATLAB (https://www.mathworks.com/products/matlab.html).

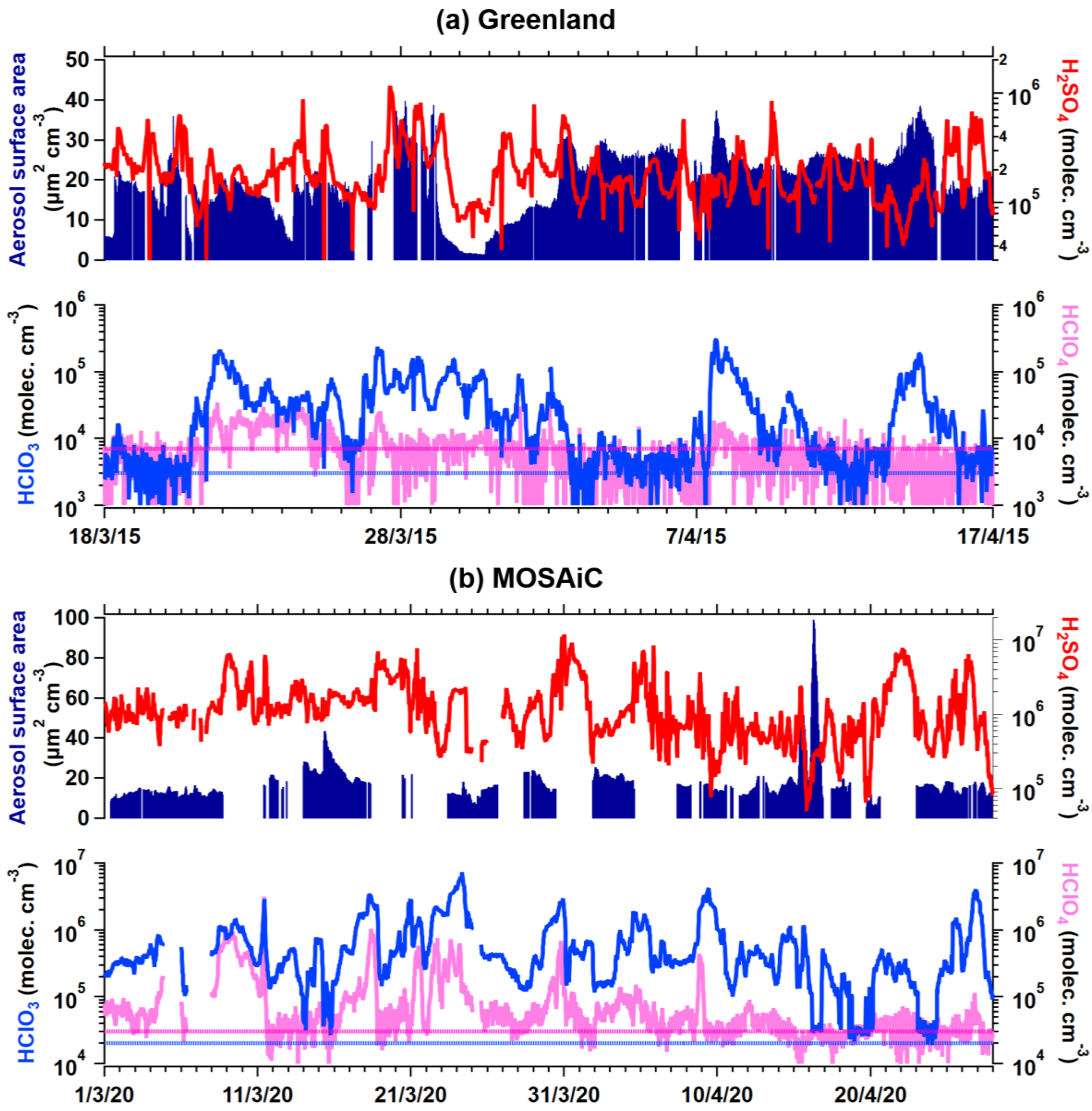

Supplementary Fig. S4 | $HClO_3$, $HClO_4$, H_2SO_4 and aerosol surface area. Expanded view between $HClO_3$, $HClO_4$ and the measured H_2SO_4 and aerosol surface area at (**a**) the Villum Research Station, Greenland (18 March to 17 April 2015) and (**b**) during the MOSAiC campaign (1 March to 28 April 2020). The gap in the aerosol surface area in the data set is either due to the removal of invalid data (i.e., particle counts too low or due to local emissions), instrument maintenance or instrumentation offline events. The dashed-line represents the detection limits for $HClO_3$ (blue) and $HClO_4$ (pink) measurements. The uncertainty of $HClO_3$ and $HClO_4$ measurements was estimated to be at least a factor of two (see Methods).

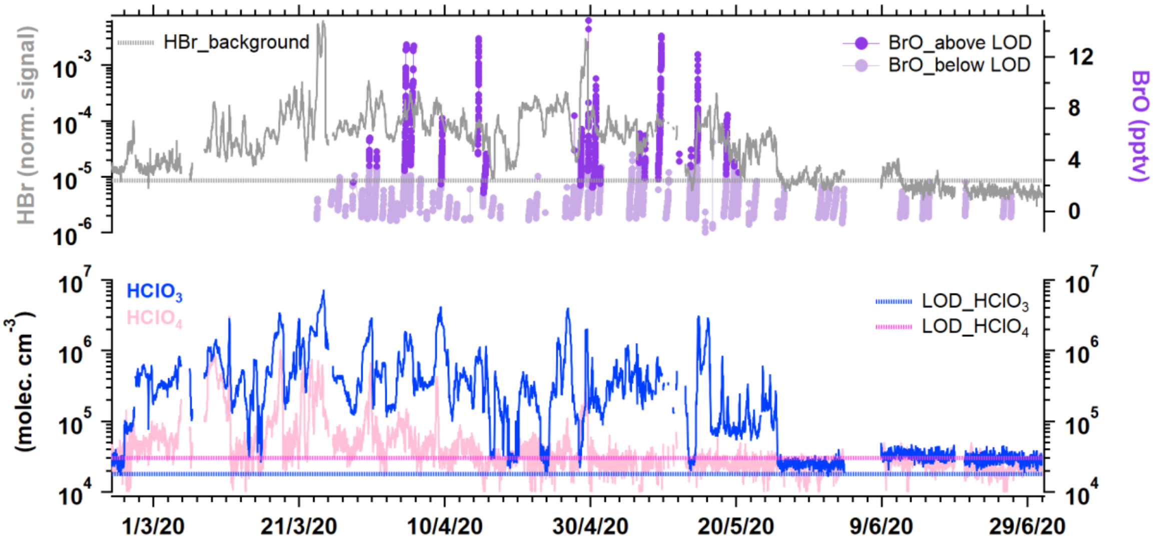

Supplementary Fig. S5 | $HClO_3$, $HClO_4$ and bromine observations during MOSAiC. The time series for ground-based BrO, HBr (Br^- signal), corresponding to the $HClO_3$ and $HClO_4$ observations during the MOSAiC campaign. Details of the ground-based BrO measurement can be found in Benavent *et al.*[7]. The dashed-line represents the detection limits (LOD) for $HClO_3$ (blue) and $HClO_4$ (pink). The grey dashed-line is the background signal obtained during the zero measurements of HBr (Br^- signal). Note that the uncertainty of $HClO_3$ and $HClO_4$ measurements was estimated to be at least a factor of two.

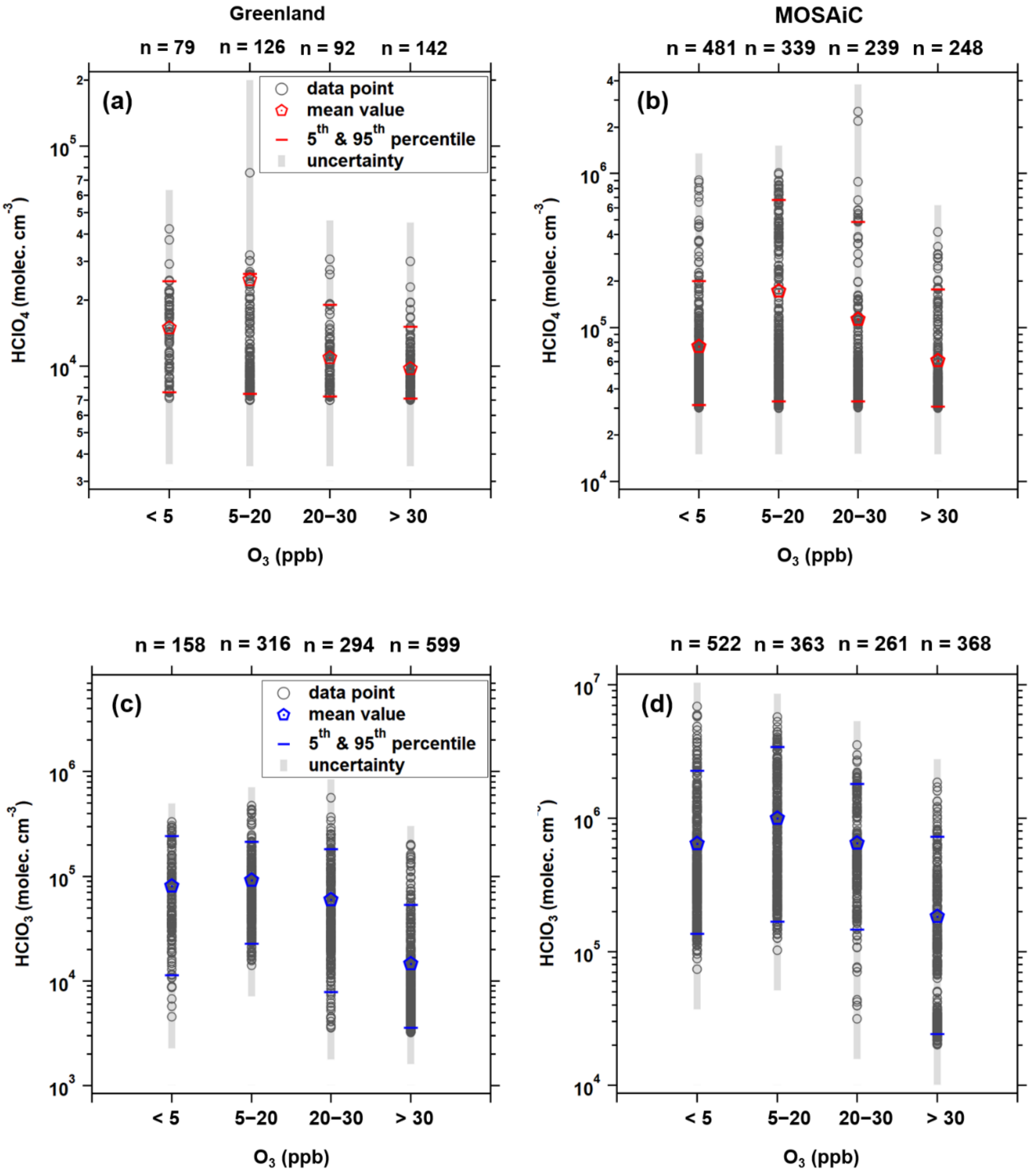

Supplementary Fig. S6 | $HClO_4$ and $HClO_3$ under different O_3 levels. Distribution of $HClO_4$ and $HClO_3$ under a different range of O_3 concentrations at (**a, b**) the Villum Research Station, Greenland; and (**c, d**) during the MOSAiC expedition from 22 February to 30 April 2020. Significant concentrations of $HClO_3$ and $HClO_4$ were frequently observed when the O_3 concentration was below 30 ppb (also refer to Fig.1 and Fig.2). Note that when O_3 is less than 5 ppb, we defined it as 'complete' depletion of O_3. In this plot, all the data points below the detection limits were removed. The uncertainty of a factor of two for the $HClO_4$ measurements has been shown in the plot (grey shaded-area).

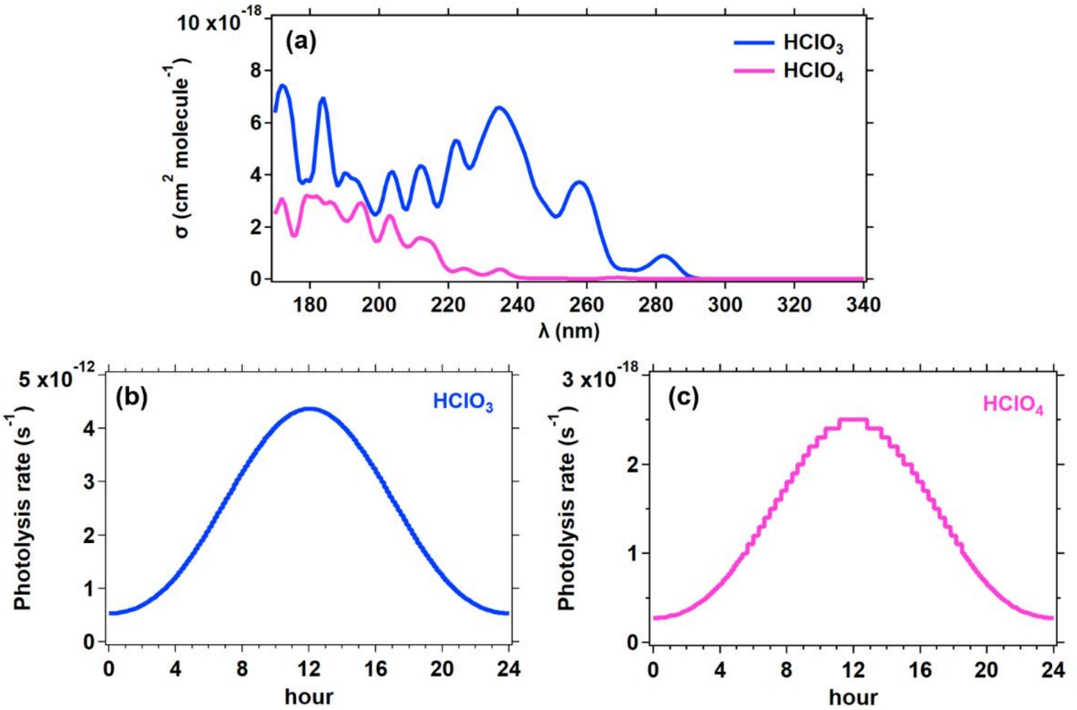

Supplementary Fig. S7 | Photolysis of $HClO_3$ and $HClO_4$. (**a**) Modelled absorption cross-section (σ) of $HClO_3$ and $HClO_4$. Photolysis rate calculated based on the absorption cross-section for (**b**) $HClO_3$ and (**c**) $HClO_4$ in the Arctic environment with a latitude of 81° 21' N (similar to the location of Villum Research Station, Greenland) on 1 May 2020.

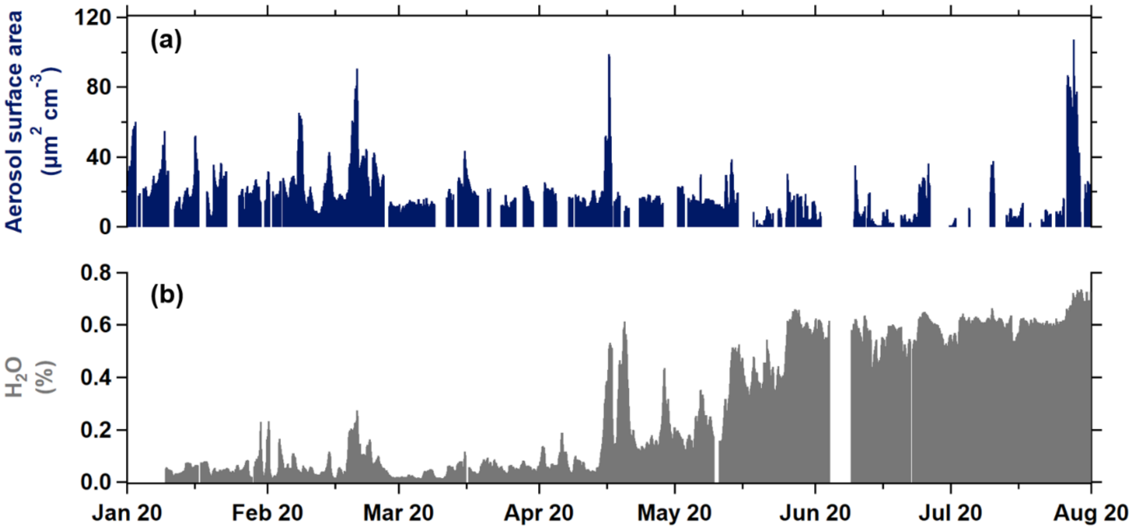

Supplementary Fig. S8 | Aerosol surface area and humidity during the MOSAiC campaign. (**a**) Aerosol surface area estimated according to the scanning mobility particle sizer (SMPS) measurements and (**b**) H_2O measured throughout the MOSAiC campaign from January to July 2020. The data of the aerosol surface area and H_2O exhibit a 30-min average of time resolution.

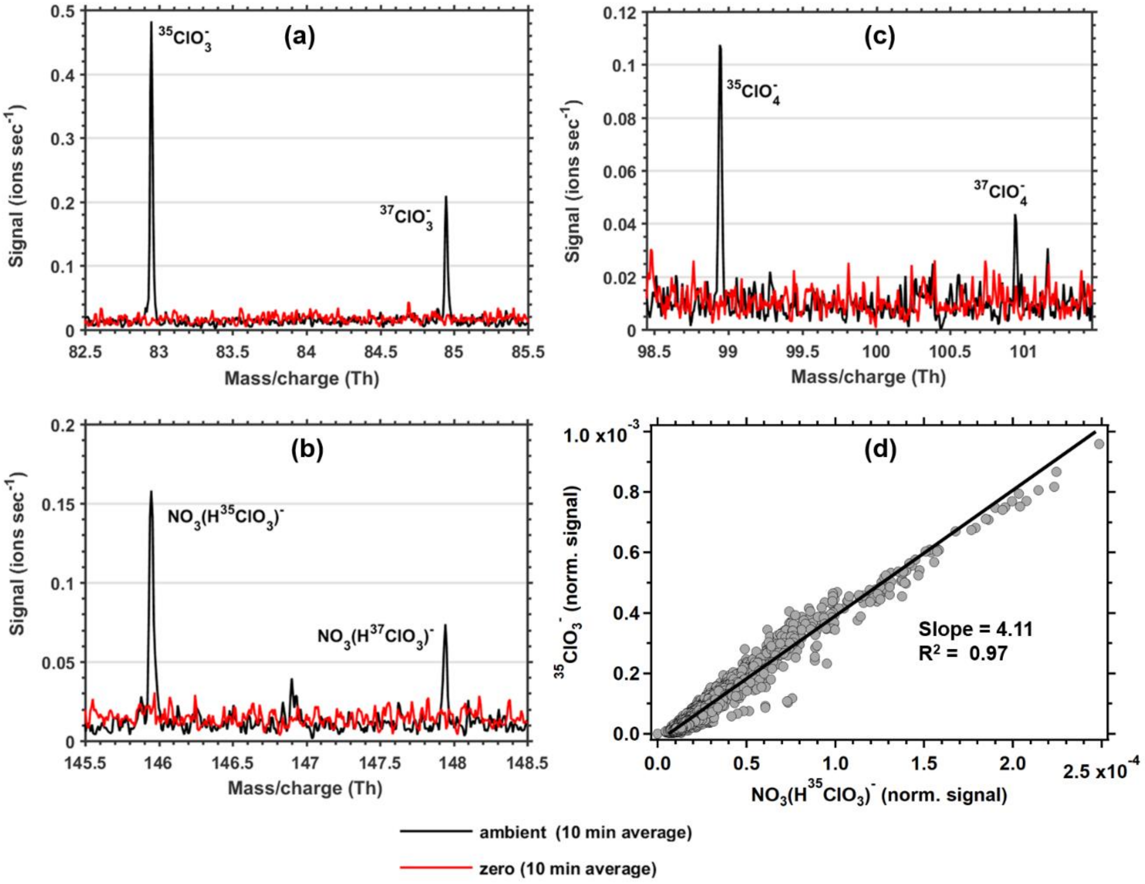

Supplementary Fig. S9 | Peak identification for $HClO_3$ and $HClO_4$. The selected mass spectra reveal the corresponding peaks (black line) and mass spectrum of the zero measurements (red line) by the nitrate CI-APi-TOF for (**a**) deprotonated ions of $HClO_3$ and ClO_3^-, (**b**) clusters of $HClO_3 \cdot NO_3^-$ and (**c**) deprotonated ions of $HClO_4$ and ClO_4^-. Note that the $HClO_4 \cdot NO_3^-$ cluster is not detected in the mass spectrum. There are absences of peaks for $HClO_3$ and $HClO_4$ during the zero measurement (also refer to Supplementary Fig. S14). (**d**) The scatter plot of $^{35}ClO_3^-$ versus $NO_3(H^{35}ClO_3)^-$ normalized signal, obtained during the MOSAiC from 22 February to 30 April 2020. The slope is 4.11 with R^2 of 0.97.

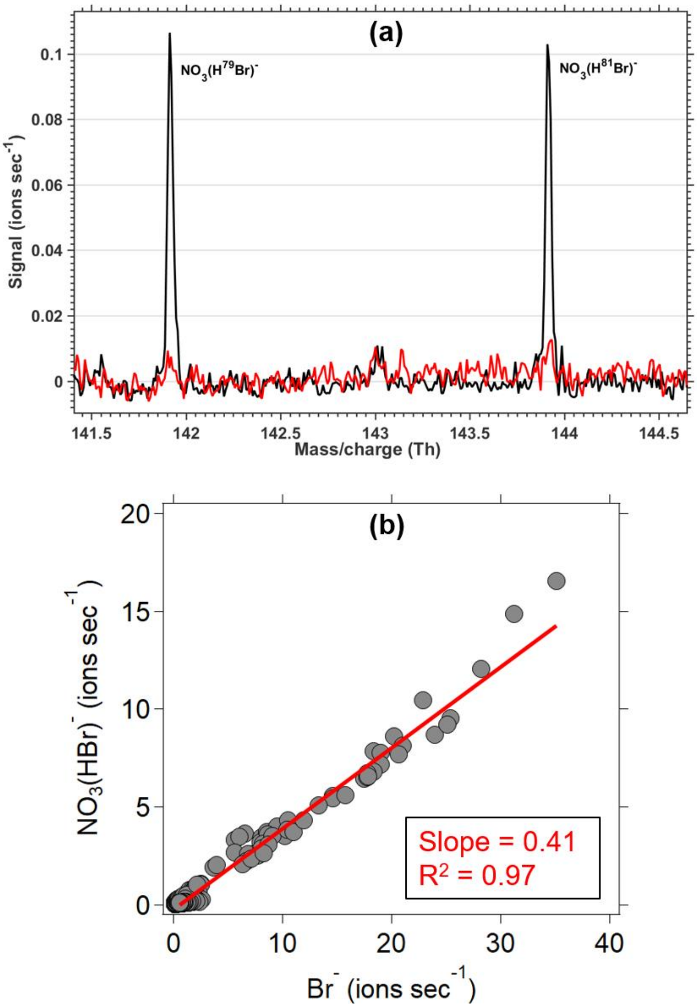

Supplementary Fig. S10 | Observation of $NO_3(HBr)^-$ signal. (**a**) The peak of $NO_3(H^{79}Br)^-$ together with its isotope peak ($NO_3(H^{81}Br)^-$), compared to the zero measurement (red line), detected by nitrate CI-APi-TOF. (**b**) An example of the $NO_3(HBr)^-$ versus Br^- data obtained during the MOSAiC from 18 to 30 April 2020.

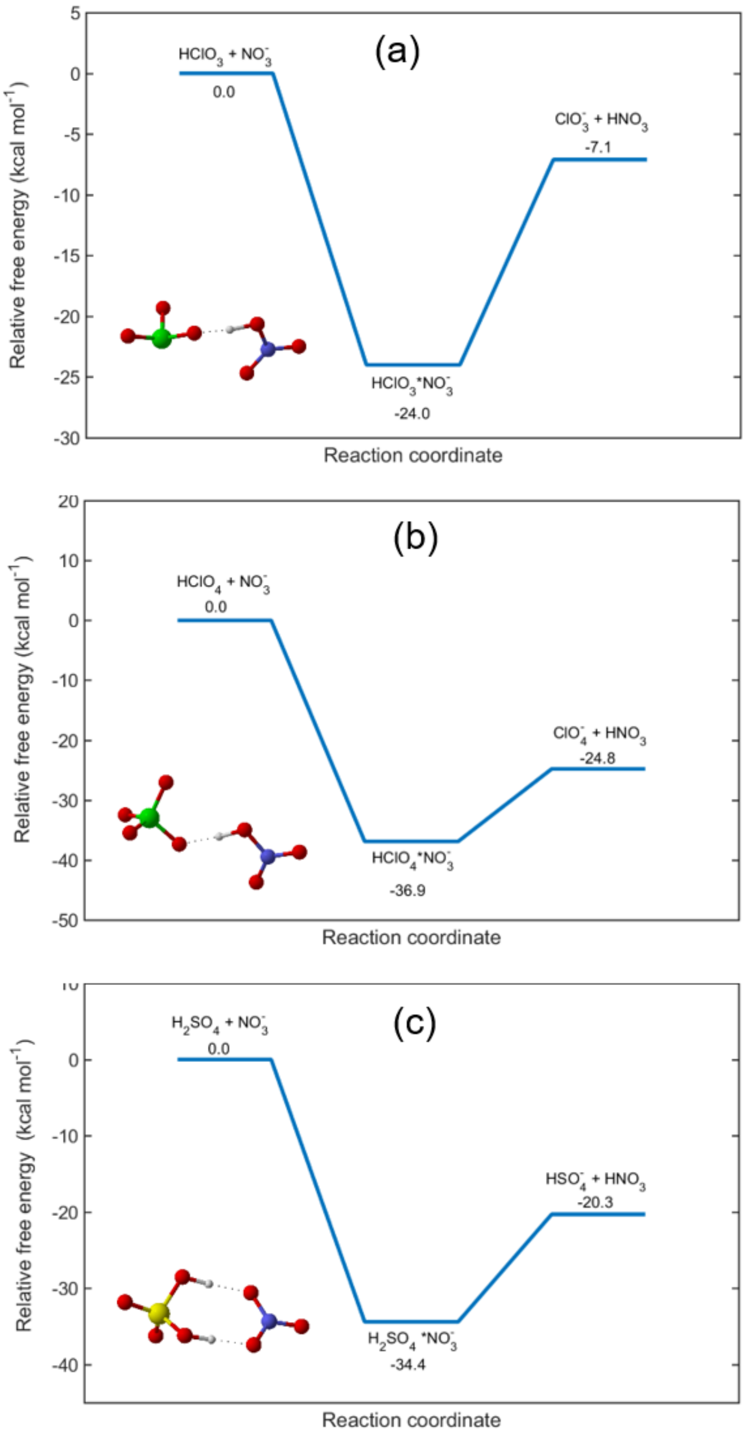

Supplementary Fig. S11 | Cluster formation free energy. Potential energy surface (PES) indicating the cluster formation free energies for (**a**) NO_3^- clustering of $HClO_3$, (**b**) NO_3^- clustering of $HClO_4$, and (**c**) NO_3^- clustering of H_2SO_4. The cluster geometries are included: green = chlorine, red = oxygen, white = hydrogen, blue = nitrogen, and yellow = sulfur atoms.

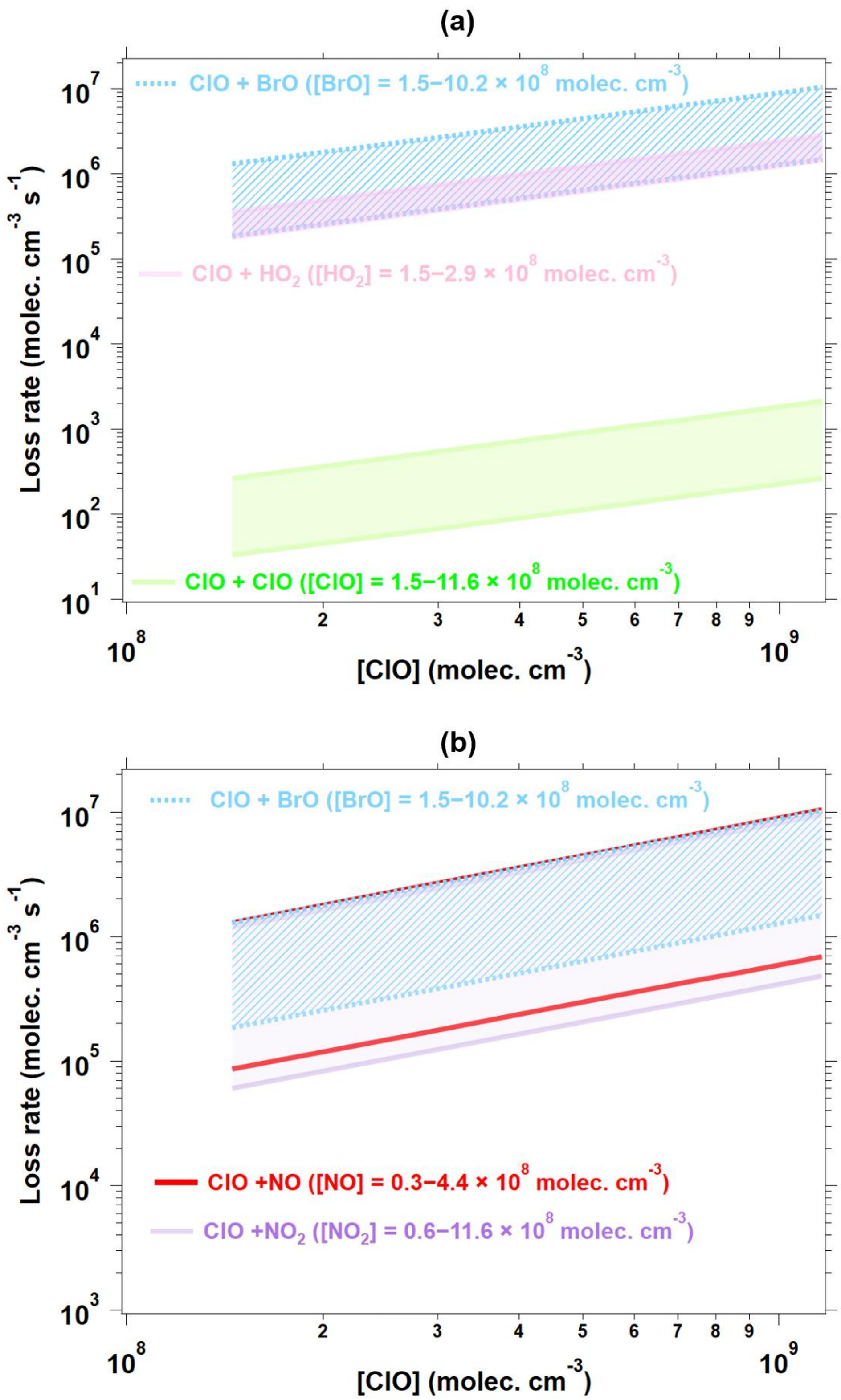

Supplementary Fig. S12 | Loss rates of ClO. The calculated rates of (**a**) ClO consumed by BrO (blue dash line), HO_2 (pink) and ClO (green). (**b**) Comparison of the rates of ClO + BrO with rates of ClO + NO and ClO+NO_2. Note that the shaded area represents the range of loss rate calculated from different levels of BrO (5−35 ppt), HO_2 (5−10 ppt), and ClO (5−40 ppt) reported in the spring ozone depletion events in the Arctic[2-6,8-11]. The concentrations of NO (1−15 ppt) and NO_2 (2−40 ppt) were adopted from the ATom flight measurements in the Arctic[13].

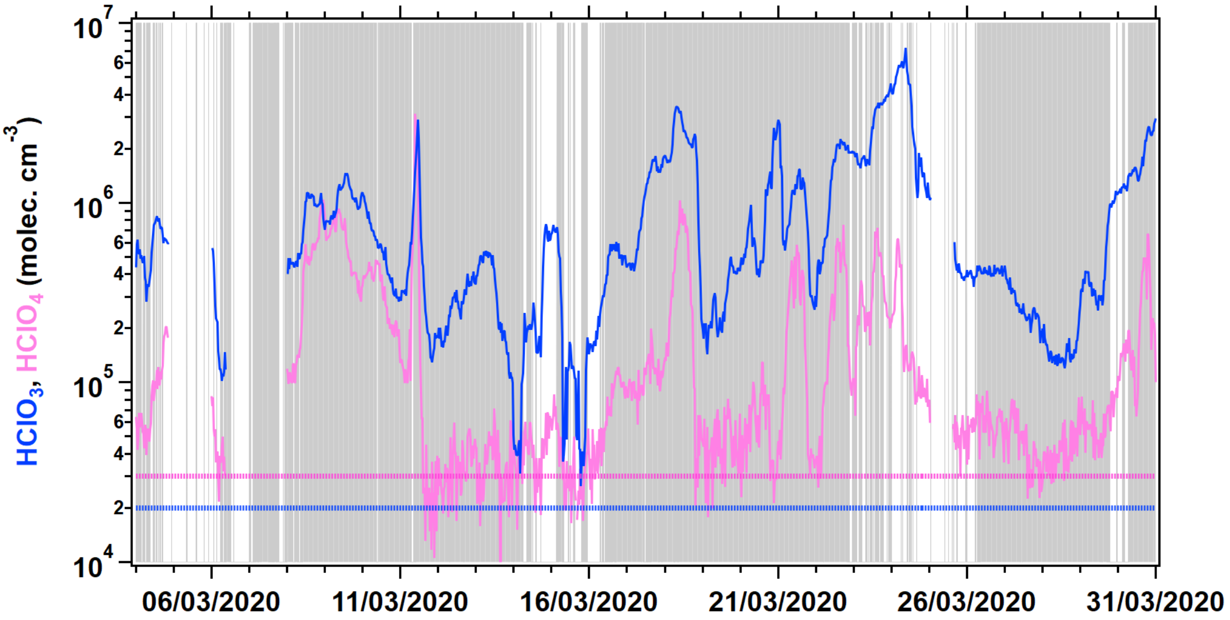

Supplementary Fig. S13 | Expanded view of $HClO_3$ and $HClO_4$ levels during the measurement period with potential influence of ship pollution in the MOSAiC campaign. The grey shaded area (with a time resolution of 1 min) denotes the period where the particle number concentration measurements were impacted by the ship pollution, as defined in Beck *et al.*[37]. This figure is indicative only and does not necessarily reflect the percentage of clean data points collected by other instruments during the expedition. There is, however, a relatively high probably that NO_x levels were elevated during these periods. The dashed-line represents the detection limits for $HClO_3$ (blue) and $HClO_4$ (pink) measurements. Note that the uncertainty of $HClO_3$ and $HClO_4$ measurements was estimated to be at least a factor of two.

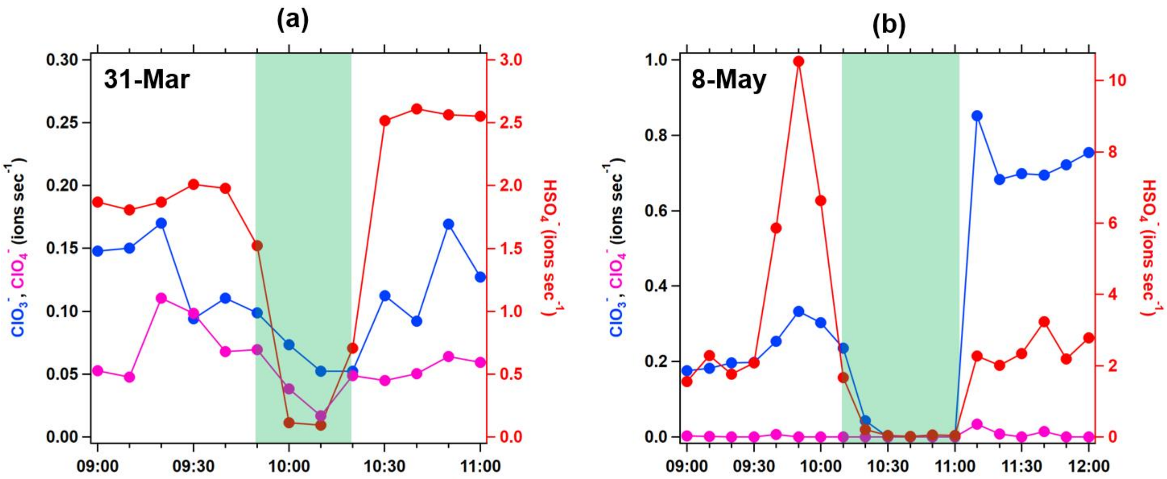

Supplementary Fig. S14 | Zero measurements in Greenland. Example of zero measurements in Greenland conducted on (**a**) 31 March 2015 and (**b**) 8 May 2015. The shaded area (light green) is to the zero measurement period. The ClO_3^-, ClO_4^- and HSO_4^- signals correspond to $HClO_3$, $HClO_4$ and H_2SO_4, respectively.

Supplementary References

1 Atkinson, R. *et al.* Evaluated kinetic and photochemical data for atmospheric chemistry: Volume III gas phase reactions of inorganic halogens. *Atmos. Chem. Phys.* **7**, 981-1191 (2007).

2 Pöhler, D., Vogel, L., Frieß, U. & Platt, U. Observation of halogen species in the Amundsen Gulf, Arctic, by active long-path differential optical absorption spectroscopy. *Proc. Natl. Acad. Sci. U.S.A* **107**, 6582 (2010).

3 Martinez, M., Arnold, T. & Perner, D. The role of bromine and chlorine chemistry for arctic ozone depletion events in Ny-Ålesund and comparison with model calculations. *Ann. Geophys.* **17**, 941-956 (1999).

4 Liao, J. *et al.* Characterization of soluble bromide measurements and a case study of BrO observations during ARCTAS. *Atmos. Chem. Phys.* **12**, 1327-1338 (2012).

5 Tuckermann, M. *et al.* DOAS-observation of halogen radical-catalysed Arctic boundary layer ozone destruction during the ARCTOC-campaigns 1995 and 1996 in Ny-Ålesund, Spitsbergen. *Tellus B: Chem. Phys. Meteorol.* **49**, 533-555 (1997).

6 Wang, S. *et al.* Direct detection of atmospheric atomic bromine leading to mercury and ozone depletion. *Proc. Natl. Acad. Sci. U.S.A* **116**, 14479 (2019).

7 Benavent, N. *et al.* Substantial contribution of iodine to Arctic ozone destruction. *Nat. Geosci.* **15**, 770-773 (2022).

8 Thompson, C. R. *et al.* Interactions of bromine, chlorine, and iodine photochemistry during ozone depletions in Barrow, Alaska. *Atmos. Chem. Phys.* **15**, 9651-9679 (2015).

9 Custard, K. D., Pratt, K. A., Wang, S. & Shepson, P. B. Constraints on Arctic atmospheric chlorine production through measurements and simulations of Cl_2 and ClO. *Environ. Sci. Technol.* **50**, 12394-12400 (2016).

10 Edwards, P. *et al.* Hydrogen oxide photochemistry in the northern Canadian spring time boundary layer. *J. Geophys. Res. Atmos.* **116**, D22306 (2011).

11 Thompson, C. R. *et al.* Bromine atom production and chain propagation during springtime Arctic ozone depletion events in Barrow, Alaska. *Atmos. Chem. Phys.* **17**, 3401-3421 (2017).

12 Bourgeois, I. *et al.* Comparison of airborne measurements of NO, NO_2, HONO, NO_y, and CO during FIREX-AQ. *Atmos. Meas. Tech.* **15**, 4901-4930 (2022).

13 Ryerson, T. B., Thompson, C. R., Peischl, J. & Bourgeois, I. In Situ Measurements from NOAA nitrogen oxides and ozone (NO_yO_3) instrument, *ORNL Distributed Active Archive Center* (2019).

14 Zhu, R. S. & Lin, M. C. Ab initio chemical kinetics for ClO reactions with HO_x, ClO_x and NO_x (x=1,2): A review. *Comput. Theor. Chem.* **965**, 328-339 (2011).

15 Hornbrook, R. S. *et al.* Arctic springtime observations of volatile organic compounds during the OASIS-2009 campaign. *J. Geophys. Res. Atmos.* **121**, 9789-9813 (2016).

16 Stone, D., Whalley, L. K. & Heard, D. E. Tropospheric OH and HO_2 radicals: field measurements and model comparisons. *Chem. Soc. Rev.* **41**, 6348-6404 (2012).

17 Liao, J. *et al.* High levels of molecular chlorine in the Arctic atmosphere. *Nat. Geosci.* **7**, 91-94 (2014).

18 Mielke, L. H. *et al.* Heterogeneous formation of nitryl chloride and its role as a nocturnal NO_x reservoir species during CalNex-LA 2010. *J. Geophys. Res. Atmos.* **118**, 10,638-610,652 (2013).

19 Wavefunction, Inc. Spartan'16 version 1.1.9. (Irvine, CA).

20 Chai, J.-D. & Head-Gordon, M. Long-range corrected hybrid density functionals with damped atom–atom dispersion corrections. *Phys. Chem. Chem. Phys.* **10**, 6615-6620 (2008).

21 Kendall, R. A., Dunning, T. H. & Harrison, R. J. Electron affinities of the first-row atoms revisited. Systematic basis sets and wave functions. *J. Chem. Phys.* **96**, 6796-6806 (1992).

22 Frisch, M. *et al.* Gaussian 16, Revision C. 01 **(Gaussian Inc.)** (2016).

23 Riplinger, C. & Neese, F. An efficient and near linear scaling pair natural orbital based local coupled cluster method. *J. Chem. Phys.* **138**, 034106 (2013).

24 Neese, F. The ORCA program system. *WIREs Comput. Mol. Sci.* **2**, 73-78 (2012).

25 Saiz-Lopez, A. *et al.* Gas-phase photolysis of Hg(I) radical species: A new atmospheric mercury reduction process. *J. Am. Chem. Soc.* **141**, 8698-8702 (2019).

26 Saiz-Lopez, A. *et al.* Photoreduction of gaseous oxidized mercury changes global atmospheric mercury speciation, transport and deposition. *Nat. Commun.* **9**, 4796 (2018).

27 Carmona-García, J. *et al.* Photochemistry and non-adiabatic photodynamics of the HOSO radical. *J. Am. Chem. Soc.* **143**, 10836-10841 (2021).

28 Carmona-García, J. *et al.* Photochemistry of $HOSO_2$ and SO_3 and implications for the production of sulfuric acid. *J. Am. Chem. Soc.* **143**, 18794-18802 (2021).

29 Francés-Monerris, A. *et al.* Photodissociation mechanisms of major mercury(II) species in the atmospheric chemical cycle of mercury. *Angew. Chem. Int. Ed.* **59**, 7605-7610 (2020).

30 Barbatti, M. *et al.* NEWTON-X: a package for Newtonian dynamics close to the crossing seam. *Dynamics* **16** (2007).
31 Roca-Sanjuán, D., Aquilante, F. & Lindh, R. Multiconfiguration second-order perturbation theory approach to strong electron correlation in chemistry and photochemistry. *Wiley Interdiscip. Rev. Comput. Mol. Sci.* **2**, 585-603 (2012).
32 Aquilante, F. *et al.* Modern quantum chemistry with [Open]Molcas. *J. Chem. Phys.* **152**, 214117 (2020).
33 Ghigo, G., Roos, B. O. & Malmqvist, P.-Å. A modified definition of the zeroth-order Hamiltonian in multiconfigurational perturbation theory (CASPT2). *Chem. Phys. Lett.* **396**, 142-149 (2004).
34 Forsberg, N. & Malmqvist, P.-Å. Multiconfiguration perturbation theory with imaginary level shift. *Chem. Phys. Lett.* **274**, 196-204 (1997).
35 Beck, L. J. *et al.* Differing mechanisms of new particle formation at two Arctic sites. *Geophys. Res. Lett.* **48**, e2020GL091334 (2021).
36 Stein, A. F. *et al.* NOAA's HYSPLIT Atmospheric Transport and Dispersion Modeling System. *Bull. Am. Meteorol. Soc.* **96**, 2059-2077 (2015).
37 Beck, I. *et al.* Automated identification of local contamination in remote atmospheric composition time series. *Atmos. Meas. Tech.* **15**, 4195-4224 (2022).
